# Persistent Activation of Autophagy After Cisplatin Nephrotoxicity Promotes Renal Fibrosis and Chronic Kidney Disease

**DOI:** 10.3389/fphar.2022.918732

**Published:** 2022-05-30

**Authors:** Ying Fu, Yu Xiang, Wenwen Wu, Juan Cai, Chengyuan Tang, Zheng Dong

**Affiliations:** ^1^ Department of Nephrology, Hunan Key Laboratory of Kidney Disease and Blood Purification, The Second Xiangya Hospital at Central South University, Changsha, China; ^2^ Department of Cellular Biology and Anatomy, Medical College of Georgia at Augusta University and Charlie Norwood VA Medical Center, Augusta, GA, United States

**Keywords:** autophagy, cisplatin, kidney injury and repair, renal fibrosis, profibrotic growth factor

## Abstract

Autophagy, a highly conserved catabolic pathway in eukaryotic cells, contributes to the maintenance of the homeostasis and function of the kidney. Upon acute kidney injury (AKI), autophagy is activated in renal tubular cells to act as an intrinsic protective mechanism. However, the role of autophagy in the development of chronic kidney pathologies including renal fibrosis after AKI remains unclear. In this study, we detected a persistent autophagy activation in mouse kidneys after nephrotoxicity of repeated low dose cisplatin (RLDC) treatment. 3-methyladenine (3-MA) and chloroquine (CQ), respective inhibitors of autophagy at the initiation and degradation stages, blocked autophagic flux and improved kidney repair in post-RLDC mice, as indicated by kidney weight, renal function, and less interstitial fibrosis. *In vitro*, RLDC induced a pro-fibrotic phenotype in renal tubular cells, including the production and secretion of pro-fibrotic cytokines. Notably, autophagy inhibitors blocked RLDC-induced secretion of pro-fibrotic cytokines in these cells. Together, the results indicate that persistent autophagy after AKI induces pro-fibrotic cytokines in renal tubular cells, promoting renal fibrosis and chronic kidney disease.

## Introduction

Nephrotoxicity is a major limiting factor in the clinical use of cisplatin, a widely used chemotherapy drug that typically results in acute kidney injury (AKI) in 30% of cancer patients (Miller et al., 2010). Even with measures to reduce the acute toxicity, such as repeated low-dose cisplatin (RLDC) therapy (Bennis et al., 2014), the patients receiving cisplatin treatment are still at risk of progressive decline in renal function and the development of chronic kidney disease (CKD) (Skinner et al., 2009). Recent studies have begun to understand the chronic effect of cisplatin nephrotoxicity by using the animal models of RLDC treatment (Torres et al., 2016; Black et al., 2018; Sharp et al., 2018; Shi et al., 2018; Fu et al., 2019; Landau et al., 2019; Sears and Siskind, 2021). For example, we showed that, after 4 weekly injections of 8 mg/kg cisplatin, mice had chronic renal damage, interstitial fibrosis, gradual decline of renal function within 6 months (Fu et al., 2019). In addition, we developed an *in vitro* model of RLDC by treating cultured renal tubular cells repeatedly low doses of cisplatin, which led to pro-fibrotic changes in these cells characterized by the production of pro-fibrotic cytokines (Fu et al., 2019).

Renal tubulointerstitial fibrosis is a common pathological feature of progressive CKD, which is characterized by excessive deposition of extracellular matrix (ECM) in the interstitial space. Interstitial fibrosis is also a pathological feature of maladaptive kidney repair following AKI, which involves a complex interaction between multiple pathways, such as inflammation, cell cycle arrest, mitochondrial damage, autophagy and senescence, to name just a few (Grgic et al., 2012; Venkatachalam et al., 2015; Basile et al., 2016; Humphreys et al., 2016). Under this condition, renal tubular cells may undergo a dramatic change to assume a secretory phenotype for the release of profibrotic cytokines, which stimulate interstitial fibroblasts for fibrosis (Geng et al., 2009; Lan et al., 2012). Multiple mechanisms, such as cycle arrest and senescence, may contribute to the phenotypic change in renal tubular cells during maladaptive kidney repair (Ferenbach and Bonventre, 2015; Venkatachalam et al., 2015; Humphreys et al., 2016; Liu B. et al., 2018).

Autophagy is a conserved lysosomal pathway for degrading cytoplasmic components, which is critical for maintaining renal homeostasis, structure, and function (Tang et al., 2020). In AKI, autophagy is activated as an intrinsic protective mechanism in renal tubular cells (Periyasamy-Thandavan et al., 2008; Jiang et al., 2012; Tang et al., 2018; Deng et al., 2021). However, the role of autophagy in kidney repair after AKI, especially its involvement in the development of interstitial fibrosis, remains unclear and highly controversial (Choi, 2020; Tang et al., 2020). In this regard, autophagy contributes to tubular atrophy, cell death and senescence during the development of chronic kidney pathologies, leading to renal fibrosis (Koesters et al., 2010; Li et al., 2010; Forbes et al., 2011; Xu et al., 2013; Baisantry et al., 2016; Livingston et al., 2016). However, autophagy may degrade extracellular matrix proteins, such as collagen type I, and thereby suppress fibrosis (Kim S. et al., 2012; Ding et al., 2014; Li et al., 2016). It is important to examine the role of autophagy in renal fibrosis by using different models.

In this study, we have investigated autophagy in maladaptive kidney repair and associated renal fibrosis after cisplatin nephrotoxicity induced by RLDC. We show that autophagy was persistently activated during maladaptive kidney repair after RLDC treatment in mice. Autophagy inhibitors given after RLDC improved kidney repair and ameliorated renal fibrosis, supporting a pathogenic role of autophagy in maladaptive kidney repair. Mechanistically, we demonstrate that autophagy in proximal tubules may promote fibrosis by synergistically inducing tubular cell death, tubular atrophy, and especially the production of pro-fibrotic factors such as CTGF, and TGFB.

## Materials and Methods

### Animals

Male C57BL/6 mice (8 weeks of age) purchased from SJA Laboratory Animal Corporation (Changsha, Hunan, China) were housed in the pathogen-free animal facility of the Second Xiangya Hospital under a 12-h light-dark cycle with free access to food and water. Mice received saline vehicle or 8 mg/kg cisplatin (Hansoh Pharma, Jiangsu, China) via intraperitoneal injection once a week for 4 weeks (Fu et al., 2019). For intervention, animals were randomly divided into four groups: saline administration group (vehicle), cisplatin + saline group, cisplatin + chloroquine (CQ) group and cisplatin + 3-methyladenine (3-MA) group (*n* = 6/group). After the last cisplatin injection, CQ (60 mg/kg/day), 3-MA (20 mg/kg/day) or saline was administered to mice by intraperitoneal injection for seven consecutive days. Both chloroquine (C6628) and 3-methyladenine (M9281) were purchased from Sigma-Aldrich. Animals were sacrificed at 1 week, 1 month, or 6 months after the last cisplatin injection to collect samples. The kidneys were fresh frozen in liquid nitrogen or fixed in 10% neutral buffered formalin. All animal experiments were conducted in accordance with a protocol approved by the Institutional Animal Care and Use Committee of the Second Xiangya Hospital of Central South University.

### Cell Culture and Treatment

The Boston University mouse proximal tubular cell line (BUMPT) was originally obtained from Dr. Lieberthal (Boston University) and cultured in DMEM medium with 10% FBS and 10% streptomycin as previously (Fu et al., 2019). To observe the fibrotic phenotype and apoptosis induced by different cisplatin concentrations, BUMPT cells were subjected to four cycles of treatment with 0, 0.5, 1, 2 or 5 μM cisplatin. Each cycle consisted of 7 h of cisplatin incubation and 17 h of recovery in cisplatin-free medium. Also, to observe the changes in autophagy and fibrosis phenotypes at different time points under 2 μM cisplatin, we collected cell samples at the end of cycles 0, 1, 2, 3, and 4, respectively. For CQ or 3-MA treatment, cells were subjected to four cycles of treatment with 2 μM cisplatin, and then incubated with 20 μM chloroquine (CQ) or 10 mM 3-methyladenine (3-MA) for 17 h in cisplatin-free medium. For detection of autophagic flux in cells, BUMPT cells were transiently transfected with mRFP-GFP-LC3 (ptfLC3, Addgene plasmid 21,074). Cells were processed 24 h after transfection. RFP and GFP fluorescence images were collected by confocal microscopy (Zeiss, United States). The numbers of GFP-LC3 puncta per cell and RFP-LC3 puncta per cell were counted separately using ImageJ. The number of autophagosomes was represented by GFP dots, and the number of autolysosomes was obtained by subtracting GFP dots from RFP dots. The number of autolysosomes was divided by the total number of RFP spots to express the autophagic flux rate.

### Assessment of Serum Creatinine

Serum creatinine was measured by using a commercial kit (DICT-500) from BioAssay Systems as previously described (Tang et al., 2018). In brief, blood samples were collected for coagulation and centrifugation at room temperature to collect serum. Serum samples were added to a pre-warmed (37°C) reaction mixture and the absorbance at 510 nm was monitored kinetically at 0 and 5 min of reaction. Creatinine levels (mg/dl) were then calculated based on standard curves.

### Transcutaneous Measurement of Glomerular Filtration Rate

GFR was measured in mice by transcutaneously monitoring the clearance of FITC-labeled sinistrin. Briefly, mice were anesthetized with isoflurane through an inhalation anesthesia device. A small patch on the flank of the mice was then shaved, and the transdermal GFR monitors were adhered to the skin using a double-sided adhesive patch (MediBeacon, Mannheim, Germany). Devices were secured on the mouse by wrapping with medical tape. FITC-sinistrin (7 mg/100 g b. w.) was injected via a tail vein. Mice were placed back in their cages separately, and GFR was monitored for 1–2 h. The devices were then removed, and data were analyzed using elimination kinetics curve of FITC-sinistrin as previously described (Ellery et al., 2015; Fu et al., 2019).

### Histological Staining

Hematoxylin and eosin staining. Kidney tissues were fixed with 4% paraformaldehyde, embedded in paraffin, and cut into 4 μm sections for hematoxylin and eosin staining. The renal cortex and outer medulla were examined. The degree of morphological changes was determined by light microscopy in a blinded fashion. The following measures were chosen as an indication of morphological damage to the kidney after treatment with vehicle or cisplatin: tubular necrosis, loss of the brush border, proximal tubule degradation, tubular casts, presence of inflammatory cells, and interstitial fibrosis. These measures were evaluated on a scale of 0–4, which ranged from not present 0) to mild 1), moderate 2), severe 3), and very severe 4).

Masson trichrome staining. Masson trichrome staining was performed to evaluate collagen fibrils in renal tissues using the reagents from Servicebio (Wuhan, China). For quantification, 10-20 positive collagen-stained fields (× 100 magnification) were randomly selected from each section and analyzed by Image-Pro Plus 6.0. The ratio of the blue-stained area to the area of the entire field (glomeruli, tubule lumina, and blood vessels, if any, excluded) was assessed and expressed as a percentage of the fibrotic area.

### Immunofluorescence Staining

For immunofluorescence of LC3B, a modified immunofluorescence staining protocol was performed as previously described (35). Briefly, kidney tissue sections were deparaffinized and subjected to antigen retrieval in a buffer with EDTA. The following steps were performed using the Tyramide SuperBoost™ Kit and Alexa Fluor™ Tyramides (Thermo Fisher Scientific, United States). The slides were incubated with 3% hydrogen peroxide solution for 1 h at room temperature. After washing with PBS, they were incubated with blocking buffer for 1 h, and then exposed to 1:5,000 anti-LC3 (NB100-2220, Novus Biologicals) overnight at 4°C, followed by incubation with Alexa Fluor 594-conjugated goat anti-rabbit IgG for 1 h at room temperature. After washing with PBS, the slides were incubated in tyramide working solution and then reaction stop reagent. For quantification, 10 to 20 random fields (800 magnification) were selected for each tissue to evaluate the LC3B puncta in each tubule; For fluorescent staining of collagen I, slides were washed with PBS and fixed with cold methanol:acetone (1:1) for 10 min at room temperature. After washing, fixed cells were blocked with 10% normal goat serum for 30 min. Cells were then incubated with anti-collagen I (AF7001, Affinity, Jiangsu, China) overnight at 4°C and exposed to goat anti-rabbit IgG conjugated to Alexa Fluor 594 for 1 h at room temperature. Hoechst 33,342 was used as a counterstain of the nucleus.

### ELISA

ELISA kits of CTGF(CSB-E07877 m), TGF-β1 (CSB-E04726 months) were from CUSABIO BIOTECH (Wuhan, China). Briefly, cell specimens were centrifuged at 1,000 g for 15 min at 4°C to collect cell culture supernatants. 100 μl standard and test samples were added to the wells respectively, covered with stickers after mixing, and incubated at 37 °C for 2 h. Then 100 μl biotin-labeled antibody working solution was added to each well and incubated at 37 °C for 1 h. The plate was washed three times, and 100 μl horseradish peroxidase-labeled avidin working solution was added to each well, and incubated at 37°C for 1 h. Then, 90 μl substrate solution was added in sequence, and the color was developed at 37°C for 15–30 min in the dark. 50 μl stop solution was added to stop the reaction. The optical density (OD value) of each well was measured with a microplate reader at a wavelength of 450 nm within 5 min after the reaction was terminated. A standard curve was constructed by measuring OD of the standards. Finally, the sample concentration was calculated according to the equation of the standard curve.

### Immunoblot Analysis

Renal cortical and outer medulla tissues were lysed using 2% SDS buffer with 1% protease inhibitor cocktail (P8340, Sigma-Aldrich). Protein concentration was determined using a Pierce BCA protein assay kit (no. 23225) from Thermo Scientific. Equal amounts of proteins were separated by SDS-polyacrylamide gels and then transferred onto polyvinylidene difluoride membranes. Membranes were blocked with 5% fat-free milk or 5% BSA for 1 h and subsequently incubated with primary antibodies at 4°C overnight and secondary antibodies for 1 h at room temperature. Primary antibodies used in present study were from following sources: anti-Vimentin 5,741) and anti-cleaved Caspase-3 (Asp175) 9,661) from Cell Signaling Technology; anti-LC3B (NB100-2220) from Novus Biologicals; anti-α-Smooth Muscle Actin (ab5694) and anti-Fibronectin (ab2413) from Abcam; anti-GAPDH (10494-1-AP) from Proteintech; anti-Collagen 1 (AF7001) from Affinity; all secondary antibodies for immunoblot analysis from Thermo Fisher Scientific. Antigen-antibody complexes on the membranes were detected with an enhanced chemiluminescence kit from Thermo Scientific. For quantification, protein bands were analyzed with ImageJ software.

### Quantitative Real-Time PCR

Total RNA from kidney tissues and cells was extracted with TRIzol reagents from CWBIO (Jiangsu, China) according to the manufacturer’s protocol. cDNA was synthesized using Taqman RT reagents (TaKaRa, Japan). Quantitative real-time PCR was performed using the TB Green Premix Ex Taq II reagent (TaKaRa) on a LightCycler96 Real-Time PCR System. Relative expression was normalized to the expression levels of GAPDH. The primer sequences used for qPCR are shown in Table 1.

**TABLE 1:** Primer sequences used for quantitative qPCR.

Gene	Forward	Reverse
*Tgfβ*	*5′- GAG​CCC​GAA​GCG​GAC​TAC​TA-3′*	*5′-GTT​GTT​GCG​GTC​CAC​CAT​T-3′*
*Ctgf*	*5′- GAC​CCA​ACT​ATG​ATG​CGA​GCC-3′*	*5′-TCC​CAC​AGG​TCT​TAG​AAC​AGG-3′*
*Pdgfβ*	*5′-TCT​CTG​CTG​CTA​CCT​GCG​TCT​G-3′*	*5′-CGT​CTT​GCA​CTC​GGC​GAT​TAC​AG-3′*
*Gapdh*	*5′-AGG​TCG​GTG​TGA​ACG​GAT​TTG-3′*	*5′-GGG​GTC​GTT​GAT​GGC​AAC​A-3′*

### Statistics

All *in vivo* qualitative data are representative of at least six individual animals and *in vitro* qualitative data are representative of at least five independent experiments. Morphological analysis was performed in a blinded fashion. Quantitative data are presented as mean ± SD. Statistical analysis was performed using GraphPad Prism seven software. The statistical differences of multiple groups were determined by performing multiple comparisons with ANOVA followed by Tukey’s posttests, while statistical differences between two groups were determined by two-tailed unpaired or paired Student’s *t* test. The value of *p* < 0.05 was considered significantly different.

## Results

### Persistent Autophagy Activation in Kidneys After RLDC Treatment in Mice

**FIGURE 1:**
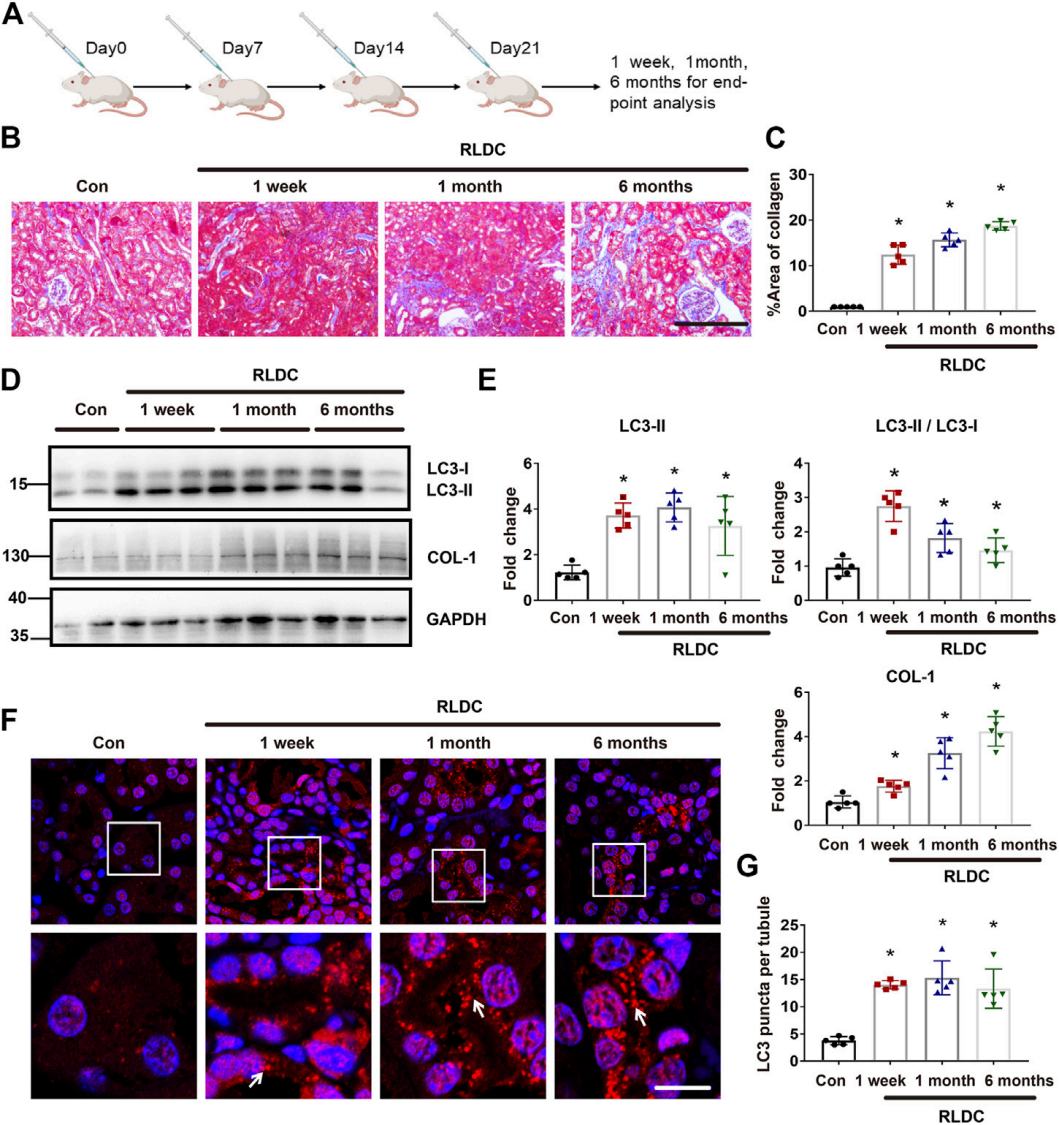
Persistent autophagy activation in kidneys after RLDC treatment in mice. Male C57BL/6 mice were injected weekly with 8 mg/kg cisplatin for 4 weeks (RLDC) or with saline as control (Con) to collect samples at 1 week, 1 month and 6 months later. **(A)** The schematic representation of the RLDC model regimen. **(B)** Masson staining of kidney tissues. (*n* = 5, bar = 50 μm). **(C)** Statistical analysis of the area of collagen deposition in Masson staining. **(D)** Representative immunoblots of LC3, COL-1 and GAPDH (loading control) in kidney tissues (*n* = 5). **(E)** Densitometry of LC3II, LC3II/LC3I and COL-1. The experiments were normalized according to GAPDH expression. The protein level of control group (Con) was arbitrarily set as 1, and the signals of other conditions were normalized with the control group to indicate their protein fold changes. **(F)** Representative images of immunofluorescence staining of LC3B. (*n* = 5). **(G)** Statistical analysis of the number of LC3B spots per renal tubule. All quantitative data are expressed as mean ± SEM. *p < 0.05 vs the control group (Con).

C57BL/6 mice were subjected to RLDC treatment that included 4 weekly injections of 8 mg/kg cisplatin (Figure 1A). Consistent with our previous work (Fu et al., 2019), we detected renal interstitial fibrosis at 1 week, 1 month, and 6 months after RLDC treatment by Masson staining (Figures 1B,C) and immunoblot analysis of collagen-I (COL-1), a fibrosis marker protein (Figures 1D,E). Interestingly, we detected autophagy activation at all these time points after RLDC treatment (Figures 1D,E). At 1 week after RLDC treatment, the autophagy marker LC3B-II increased in renal tissue to approximately 4 times over control, and was maintained at this high level at 1 and 6 months (Figures 1D,E). In immunofluorescence analysis, control mouse kidneys showed ∼4 LC3-positive spots per proximal tubule, which was increased ∼15 at 1 week, 1 month, and 6 months after RLDC treatment (Figures 1F,G). These results indicate that autophagy is persistently induced during maladaptive kidney repair after cisplatin nephrotoxicity.

### Pharmacologic Inhibition of Autophagy Alleviates Renal Dysfunction and Tubular Damage in Post-RLDC Kidneys

**FIGURE 2:**
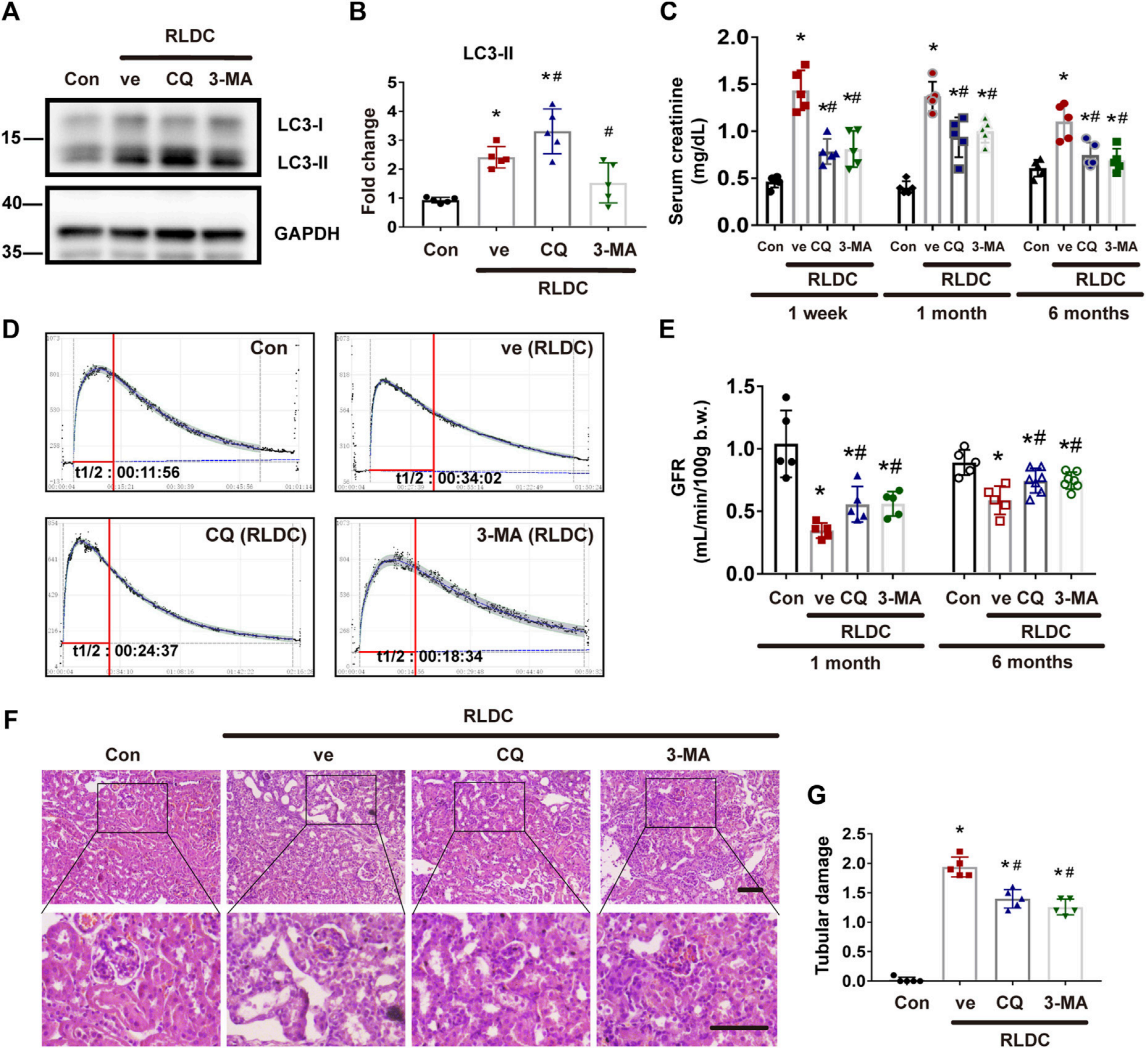
Pharmacologic inhibition of autophagy alleviates renal dysfunction and tubular damage in post-RLDC kidneys. Male C57BL/6 mice were injected weekly with 8 mg/kg cisplatin for 4 weeks (RLDC) or with saline as control (Con). After the final dose, the mice were injected with 60 mg/kg/day chloroquine (CQ), 20 mg/kg/day 3-methyladenine (3-MA), or saline as vehicle solution (ve) for 7 days. **(A)** Representative immunoblots of LC3-I, LC3-II and GAPDH (loading control) in kidney tissues (*n* = 5). **(B)** Densitometry of LC3II. The experiments were normalized according to GAPDH expression. The protein level of control group (Con) was arbitrarily set as 1, and the signals of other conditions were normalized with the control group to indicate their protein fold changes. **(C)** Effect of autophagy inhibitor on serum creatinine at 1 week, 1 month and 6 months after RLDC treatment. (*n* = 5). **(D)** Representative tracing curves of FITC-sinistrin clearance in mice. (*n* = 5). **(E)** GFR measurement by transcutaneously monitoring FITC-sinistrin clearance (*n* = 5). **(F)** Representative histology images of hematoxylin-eosin staining of kidney tissues in renal cortex and outer medulla. (*n* = 5, bar = 50 μm). **(G)** Pathological score of tubular damage. Quantitative data are expressed as mean ± SEM. *p < 0.05 vs the control group (Con), # *p* < 0.05 vs. (RLDC + vehicle) group.

To delineate the role of autophagy in maladaptive kidney repair, we studied the effects of two pharmacological inhibitors of autophagy, 3-methyladenine (3-MA) and chloroquine (CQ). 3-MA is a class III phosphatidylinositol 3-kinase inhibitor that suppresses the initial step of autophagosome formation, whereas CQ is a lysosomotropic weak base that inhibits autophagosome fusion with lysosomes and autophagic degradation (Klionsky et al., 2021). We first examined LC3 by immunoblotting. As expected, CQ induced LC3-II accumulation, whereas 3-MA reduced LC3-II formation (Figures 2A,B). For renal function, compared with the normal saline group, the serum creatinine level increased by more than 2 times at 1 week after RLDC treatment. However, the serum creatinine increase in post-RLDC mice was significantly reduced by CQ and 3-MA (Figure 2C). Moreover, we measured glomerular filtration rate (GFR) by monitoring the clearance of injected FITC-sinistrin. As shown by the clearance curves, the half-life (t1/2) of excreted FITC-sinistrin was significantly prolonged after the RLDC regimen, indicative of a decline of renal function after RLDC treatment (Figure 2D). Notably, both CQ and 3-MA reduced the half-life of FITC excretion and increased the clearance in post-RLDC mice (Figure 2D). For quantification, we calculated the GFR of these mice. As shown in Figure 2E, the GFR value of post-RLDC mice was 0.4–0.5 μl/min/100 g b. w., half of the control mice, which was improved to 0.5–0.7 μl/min/100 g b. w. by CQ and 3-MA after 1-month RLDC regimen. And the GFR value of 6-months-RLDC mice was 0.5–0.7 μl/min/100 g b. w, which was improved to 0.8–0.9 μl/min/100 g b. w. by CQ and 3-MA. Moreover, post-RLDC kidneys developed a series of pathological features, including interstitial inflammation, tubular cell death, phenotypic transition of resident renal cells, proliferation and activation of fibroblasts, and excessive ECM deposition, leading to renal fibrosis (Torres et al., 2016; Black et al., 2018; Fu et al., 2019). Histological analysis by hematoxylin-eosin staining showed that at 1 month after RLDC, renal tubules were significantly damaged, manifested by extensive necrosis of proximal tubules, cast formation, and tubular cell atrophy. There were also tubular dilation and obvious expansion of the interstitium. These pathological changes were attenuated by CQ, which reduced significantly tubular atrophy and lowered tubule damage score (Figures 2F,G). Similar effects were shown for 3-MA, the other autophagy inhibitor tested. Taken together, these results suggest that persistent autophagy in the kidney may contribute to the development of renal pathologies and the decline of renal function after RLDC treatment.

### Inhibition of Autophagy Suppresses Interstitial Fibrosis After RLDC Regimen

**FIGURE 3:**
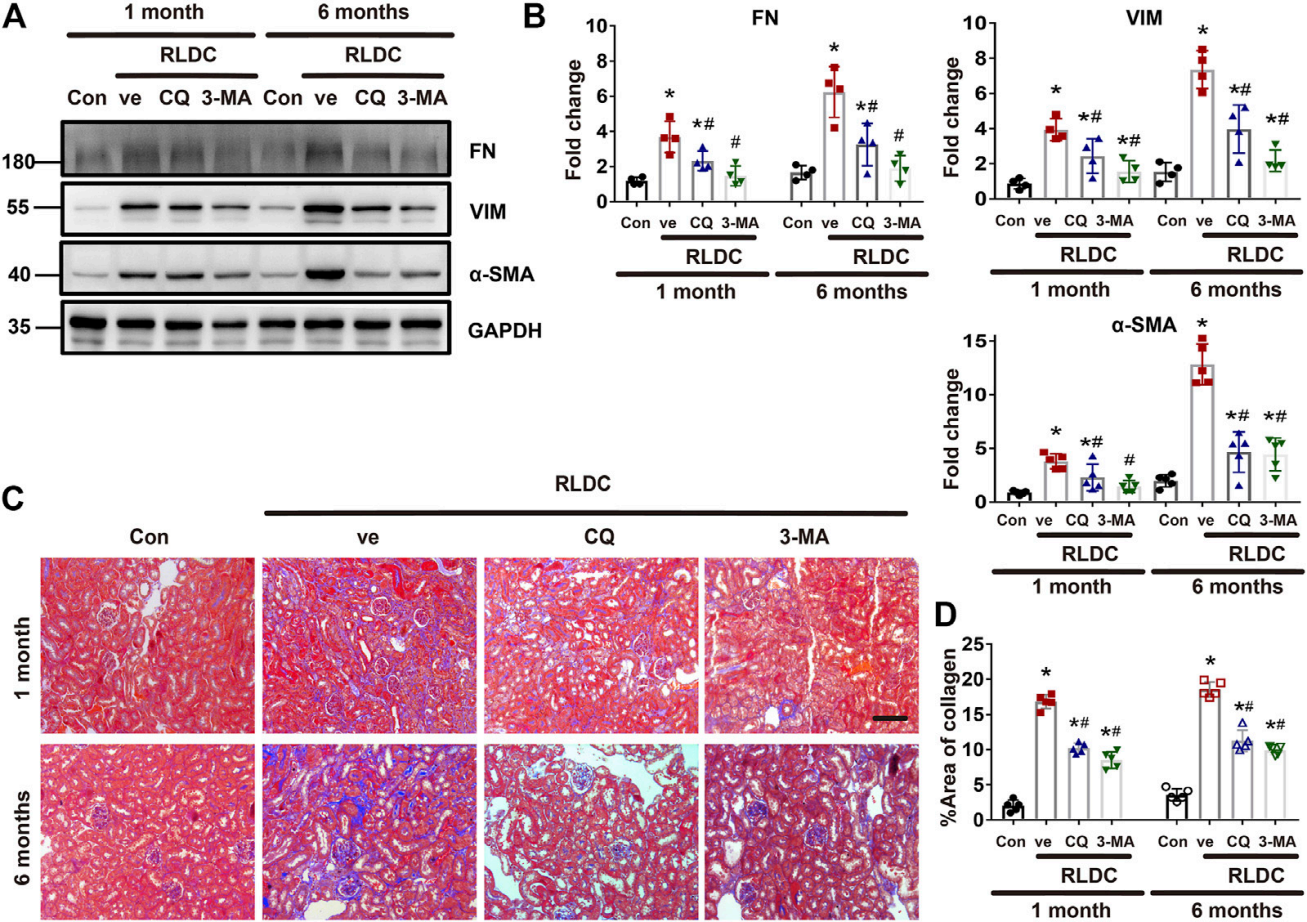
Inhibition of autophagy suppresses interstitial fibrosis after RLDC regimen. Male C57BL/6 mice were injected weekly with 8 mg/kg cisplatin for 4 weeks (RLDC) or with saline as control (Con). After the final dose, the mice were injected with 60 mg/kg/day chloroquine (CQ), 20 mg/kg/day 3-methyladenine (3-MA), or saline as vehicle solution (ve) for 7 days. **(A)** Representative immunoblots of FN, VIM, a-SMA and GAPDH (loading control) in kidney tissues (*n* = 5). **(B)** Densitometry of FN, VIM, and a-SMA. The experiments were normalized according to GAPDH expression. The protein level of control group (Con) was arbitrarily set as 1, and the signals of other conditions were normalized with the control group to indicate their protein fold changes. **(C)** Masson staining of kidney tissues. (*n* = 5, bar = 50 μm). **(D)** Statistical analysis of area of collagen deposition in Masson staining images. Data are expressed as mean ± SEM. *p < 0.05 vs the control group (Con), ^#^ *p* < 0.05 vs. (RLDC + vehicle) group.

Next, we specifically analyzed the role of autophagy in renal fibrogenesis in post-RLDC kidneys. Following RLDC treatment, renal interstitial fibrosis was significantly induced. Both CQ and 3-MA partially but significantly reduced the expression of fibrosis proteins in post-RLDC mouse kidneys, including fibronectin (FN), a-SMA and vimentin (VIM) (Figure 3A). This conclusion was substantiated by densitometry analysis of the immunoblots (Figure 3B). Masson staining further verified that CQ and 3-MA significantly reduced interstitial collagen fibril deposition in post-RLDC kidneys (Figure 3C). Morphometry showed that RLDC induced 15–17% of interstitial fibrosis at 1 month after RLDC treatment, which was reduced to 9–10% by CQ and 3-MA, and 19–20% of collagen deposition at 6 months, which was reduced to 10–12% by CQ and 3-MA (Figure 3D). The inhibitory effects of CQ and 3-MA support a role of autophagy in renal fibrogenesis during maladaptive kidney repair after cisplatin nephrotoxicity.

### RLDC Treatment Induces Autophagy and Fibrotic Phenotypes in Bumpt Cells

**FIGURE 4:**
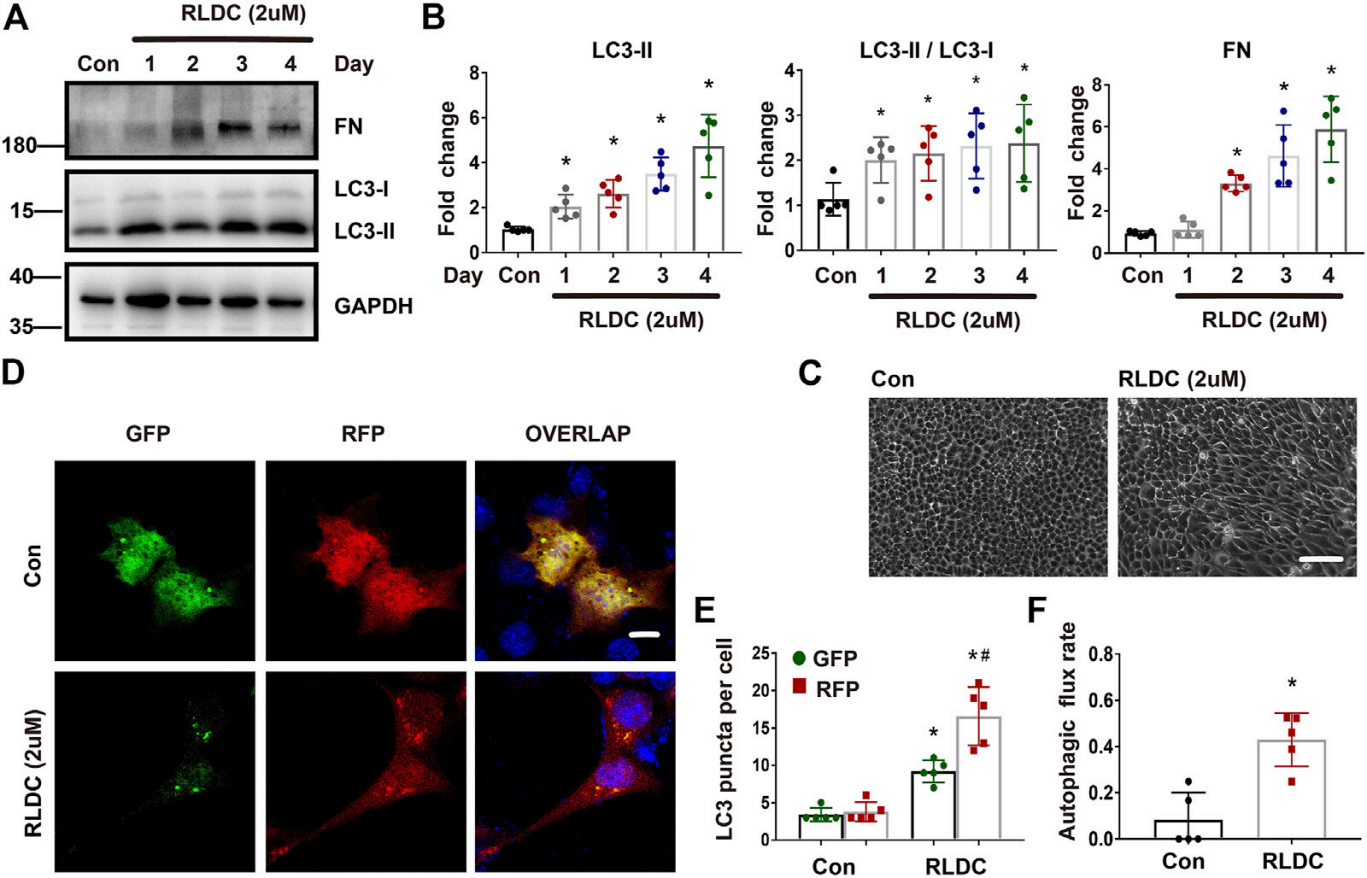
RLDC treatment induces autophagy and fibrotic phenotypes in BUMPT cells. BUMPT cells were incubated with 2 μM cisplatin for 7 h each day for 4 days (RLDC) or without cisplatin exposure (Con). **(C–F)**. **(A)** Representative immunoblots of FN, LC3-I, LC3-II and GAPDH (loading control) in cells after different cycles of cisplatin treatment. (*n* = 5). **(B)** Densitometry of FN and LC3-II, LC3II/LC3I. The experiments were normalized according to GAPDH expression. The protein level of control group (Con) was arbitrarily set as 1, and the signals of other conditions were normalized with the control group to indicate their protein fold changes. **(C)** Morphologies of control and RLDC-treated cells. (bar = 50 μm). **(D)** BUMPT cells were transiently transfected with mRFP-GFP-LC3. RFP and GFP fluorescence images were collected at 24 h after transfection by confocal microscopy. **(E)** The numbers of GFP-LC3 puncta per cell and RFP-LC3 puncta per cell were counted separately using ImageJ. The number of autophagosomes is represented by GFP dots, and the number of autolysosomes was obtained by subtracting GFP dots from RFP dots. (*n* = 5). **(F)** Autophagic flux rate. (*n* = 5). Quantitative data are expressed as mean ± SEM. *p < 0.05 vs the control group (Con).

We recently established an *in vitro* model of RLDC treatment, in which mouse kidney proximal tubular BUMPT cells were treated with low concentrations of cisplatin for 7 h daily for 4 days (Fu et al., 2019). In this model, we examined the induction of autophagy along with fibrotic changes. It was found that the fibrosis marker fibronectin (FN) was not induced until the second cycle of cisplatin exposure, whereas the autophagy marker LC-3II was elevated after the first cycle (Figures 4A,B). Morphologically, confluent BUMPT cells formed cobblestone monolayers typical of epithelium with evident cell junctions. After RLDC incubation, the cells changed to a spindle-shaped, fibroblast-like morphology (Figure 4C). We further analyzed autophagy by expressing the mRFP-GFP-LC3 tandem plasmid. Following transfection, the cells showed only minimal punctate staining under control conditions. After RLDC treatment, the cells had a large number of green GFP-LC3 and red mRFP-LC3 puncta (Figures 4D,E). The puncta with both GFP-LC3 and mRFP-LC3 signals were autophagosomes and appeared yellow in overlapping images. Once fused with lysosomes, the acid-sensitive GFP fluorescence disappeared, while the acid-insensitive RFP signal remained to indicate autolysosomes. We counted the GFP-LC3 puncta and the mRFP-LC3 puncta to calculate the autophagic flux rate (Livingston et al., 2016; Livingston et al., 2019; Klionsky et al., 2021), which increased from 8% in control cells to about 40% in RLDC-treated cells (Figure 4F). These results indicate that RLDC dynamically activate autophagy in renal tubular cells, showing the induction of autophagosome formation and the maturation into autolysosomes.

### Inhibition of Autophagy Suppresses Fibrotic Phenotype Changes in RLDC-Treated BUMPT Cells

**FIGURE 5:**
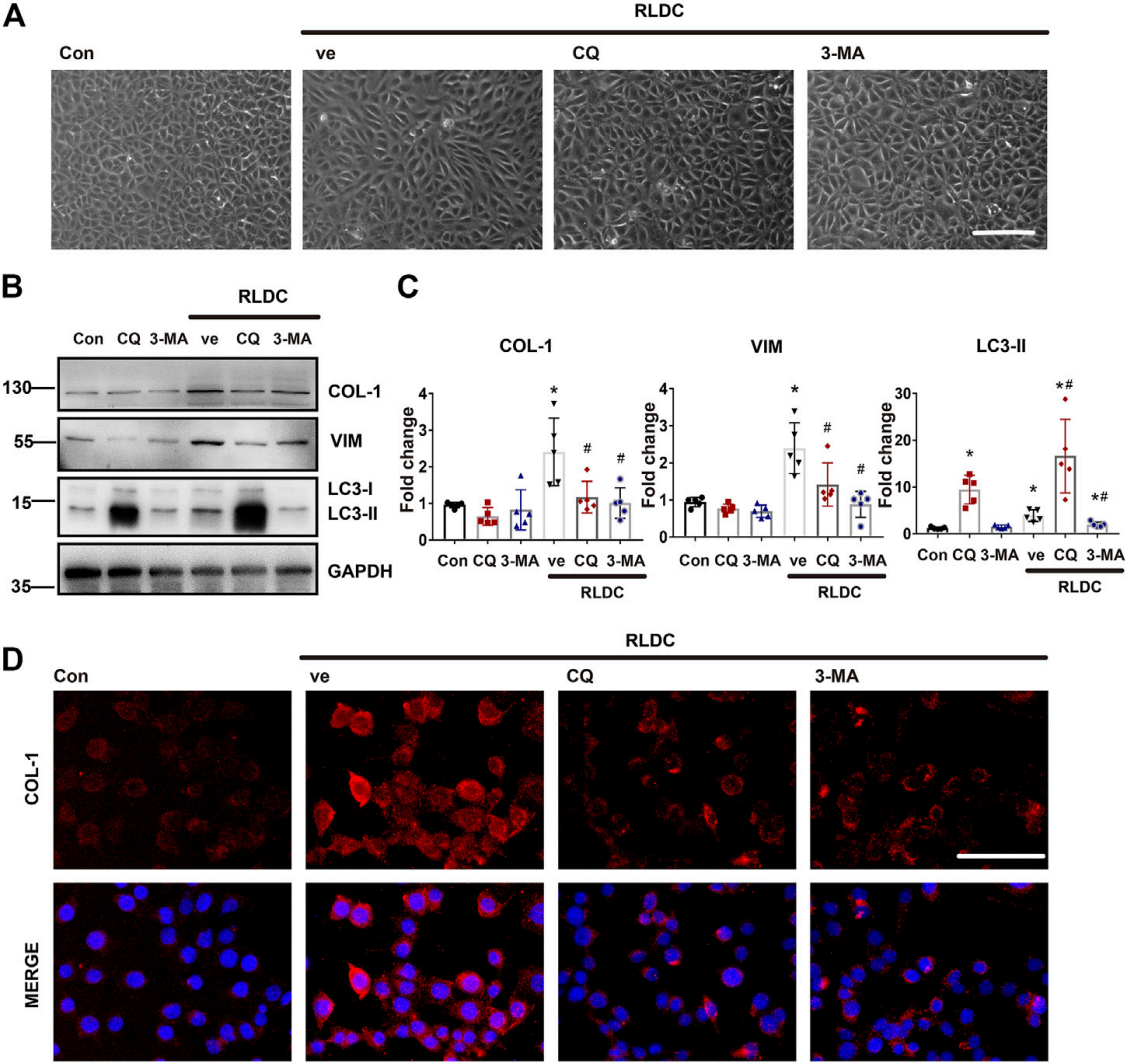
Inhibition of autophagy suppresses fibrotic phenotype changes in RLDC-treated BUMPT cells. BUMPT cells were incubated with 2 μM cisplatin for 7 h each day for 4 days (RLDC) or without cisplatin exposure (Con). After the last 7 h of cisplatin treatment, cells were incubated with 20 μM chloroquine (CQ) or 10 mM 3-methyladenine (3-MA) for 17 h in cisplatin-free medium. Cells were collected for morphological observation or biochemical analyses. **(A)** Cell morphology under light microscope after RLDC induction with or without CQ and 3-MA. (bar = 50 μm). **(B)** Representative immunoblots of COL-1, VIM, LC3-I, LC3-II and GAPDH (loading control). (*n* = 5). **(C)** Densitometry of COL-1, VIM, and LC3-II. The experiments were normalized according to GAPDH expression. The protein level of control group (Con) was arbitrarily set as 1, and the signals of other conditions were normalized with the control group to indicate their protein fold changes. **(D)** Immunofluorescence of COL-1. (n = 5, bar = 50 μm). Quantitative data are expressed as mean ± SEM. *p < 0.05 vs the control group (Con). ^#^ *p* < 0.05 vs. (RLDC + vehicle) group.

Next, we investigated the effect of autophagy inhibition on fibrotic changes *in vitro*. To do this, BUMPT cells were incubated subjected to repeated treatment with 2uM cisplatin in the absence or presence of CQ and 3-MA. Both CQ and 3-MA ameliorated RLDC-induced cellular spindle-shaped morphological changes (Figure 5A). As expected, CQ blocked autophagic flux resulting in LC3B-II increase, whereas 3-MA decreased LC3B-II production (Figure 5B). Under this condition, CQ and 3-MA reduced RLDC-induced accumulation of fibrotic marker proteins like COL-1 and VIM (Figures 5B,C). Immunofluorescence staining also confirmed that autophagy inhibited reduced collagen I in RLDC-treated cells (Figure 5D). Taken together, these results suggest that autophagy activation by RLDCs may contribute to the production of fibrotic protein markers and the induction of fibrotic phenotypes in proximal tubular cells.

### Autophagy Inhibitors Suppress the Expression and Secretion of Pro-fibrotic Growth Factors in RLDC-Treated Bumpt Cells

**FIGURE 6:**
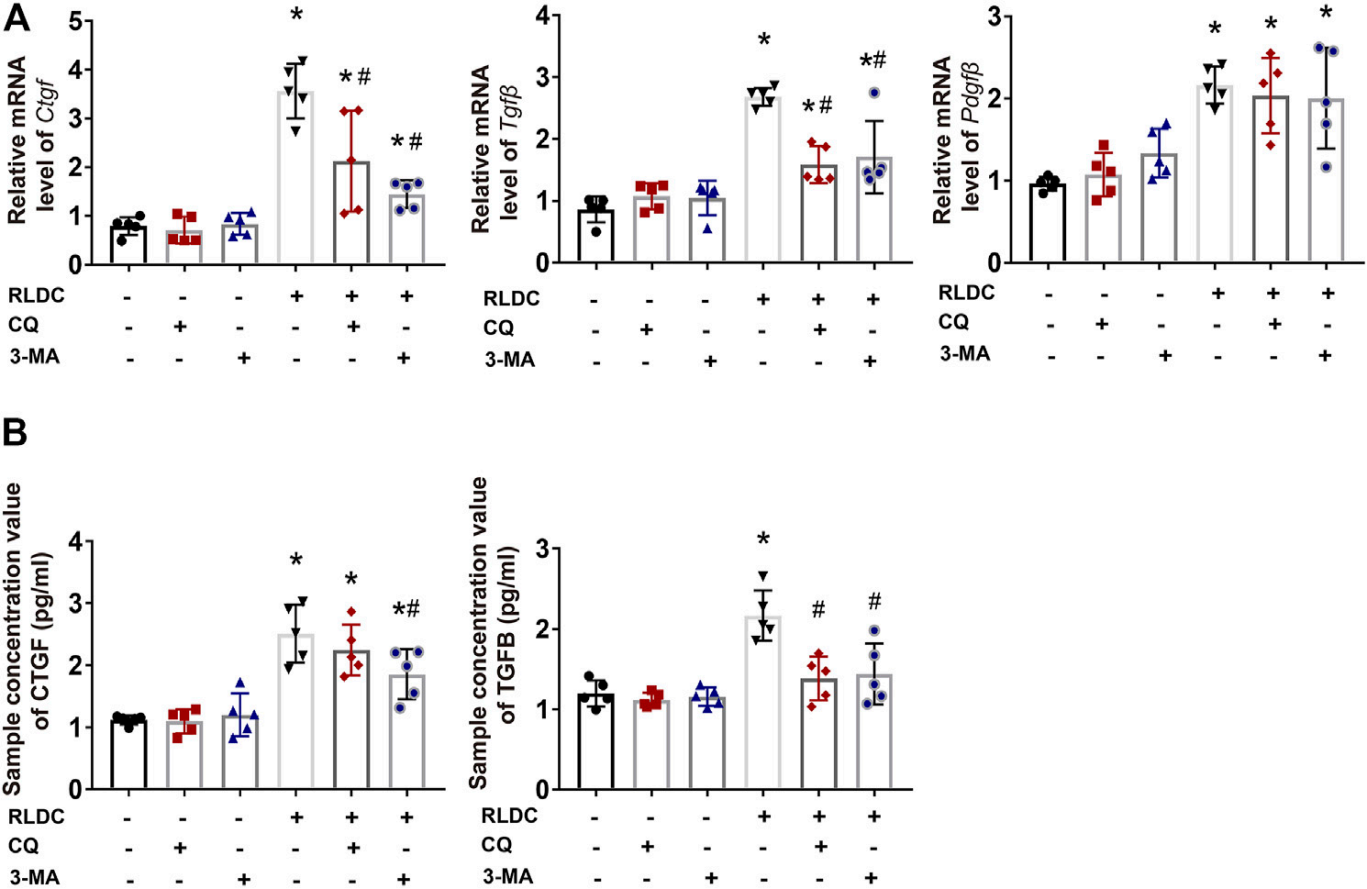
Autophagy inhibitors suppress the expression and secretion of pro-fibrotic growth factors in RLDC-treated BUMPT cells. BUMPT cells were incubated with 2 μM cisplatin for 7 h each day for 4 days (RLDC) or without cisplatin exposure (Con). After the last 7 h of cisplatin treatment, cells were incubated with 20 μM chloroquine (CQ) or 10 mM 3-methyladenine (3-MA) for 17 h in cisplatin-free medium. **(A)** The mRNA levels of *Ctgf, Tgfbβ* and *Pdgfβ* in cells quantified by qRT-PCR showing the inhibitory effect of CQ and 3-MA (*n* = 5). Data are normalized to *Gapdh* and expressed as fold change compared to controls. **(B)** CTGF and TGFB in cell culture medium detected by Elisa. (*n* = 5). Data are expressed as mean ± SEM. *p < 0.05 vs the control group (Con). ^#^ *p* < 0.05 vs. (RLDC + vehicle) group.

Mechanistically, we hypothesized that autophagy might promote renal fibrosis by inducing the expression and secretion of pro-fibrotic cytokines in kidney tubular cells. To test this possibility, after RLDC treatment, BUMPT cells were given CQ, 3-MA, or saline as control. We analyzed the expression of pro-fibrotic growth factors *Ctgf*, *Tgfβ* and *Pdgfβ* (Kok et al., 2014; Lee et al., 2015). RLDC induced mRNA expression of *Ctgf*, *Tgfβ* and *Pdgfβ*, and the former two were attenuated by both CQ and 3-MA (Figure 6A). To examine the secretion of these factors, we collected cell culture media for ELISA. The results showed that after RLDC treatment, the secretion of CTGF and TGFB by BUMPT cells increased 2-3-fold (Figure 6B). 3-MA treatment significantly reduced the secretion of CTGF and TGFB, while CQ only affected the secretion of TGFB. These results suggest that autophagy inhibitors reduce the induction of fibrotic phenotypes probably by reducing the expression and secretion of specific pro-fibrotic cytokines.

## Discussion

The role of autophagy in maladaptive kidney repair including especially renal fibrosis has been very controversial (Kim S. et al., 2012; Kim W. et al., 2012; Bernard et al., 2014; Ding et al., 2014; Li et al., 2016; Livingston et al., 2016; Shu et al., 2021). In previous work, we characterized the key features of maladaptive kidney repair in mice after RLDC treatment, including persistent inflammation, progressive interstitial fibrosis, “atubular” glomerulus and renal function decline, and further established an *in vitro* model of RLDC treatment of cultured renal tubular cells (Fu et al., 2019). Using these models, we have examined autophagy in maladaptive kidney repair and renal fibrosis after cisplatin nephrotoxicity. We detected persistent autophagy after RLDC treatment in mice and in renal tubular cells. By using pharmacological inhibitors targeting different stages of autophagy, we further demonstrated that autophagy contributes to RLDC-induced renal fibrosis *in vivo* and fibrotic phenotypes in proximal tubular cells. We also found that inhibition of autophagy reduced the degree of tubular atrophy and histopathological damage, and protected against chronic renal function decline. In the cultured renal proximal tubular cells, RLDC induced accumulation of fibrotic protein markers and secretion of pro-fibrotic growth factors, which was attenuated by autophagy inhibitors. Taken together, these results support a critical role of autophagy in maladaptive kidney repair and interstitial fibrosis following cisplatin nephrotoxicity. Mechanistically, autophagy may promote fibrosis by coordinating and inducing the production and secretion of pro-fibrotic cytokines.

We have previously shown that in nephrotoxic AKI, induction of autophagy prevents tubular cell damage and death (Periyasamy-Thandavan et al., 2008; Jiang et al., 2012; Liu J. et al., 2018; Wang et al., 2018). As for the role of autophagy in maladaptive repair, we reported the role of autophagy in unilateral ureteral obstruction (UUO) mice in 2016 (Livingston et al., 2016). In that study, autophagy was induced persistently in UUO kidneys, especially in renal tubules. Remarkably, pharmacological inhibition of autophagy with CQ and 3-MA as well as ablation of autophagy-related gene 7 (Atg7) specifically from kidney proximal tubules reduced interstitial fibrosis in UUO, indicating that tubular autophagy in renal tubules promotes renal fibrosis. Consistently, blockade of autophagy suppressed TGFB1-induced fibrotic changes in cultured renal tubular cells. Furthermore, our recent study demonstrated the reciprocal regulation between endoplasmic reticulum stress and autophagy in chronic kidney injury and fibrosis (Shu et al., 2021). Specifically, autophagy was activated during ER stress and led to renal fibrosis. These observations are consistent with our current study, which demonstrates the pro-fibrotic function of autophagy during maladaptive kidney repair after cisplatin nephrotoxicity. However, Kim and others reported that in UUO rats, inhibition of autophagy with 30 mg/kg 3-MA increased tubular apoptosis and interstitial fibrosis (Kim W. et al., 2012), an observation that was opposite to what we found. The cause of this discrepancy might be the disparity between the model species, as well as the difference in 3-MA dosing. Of note, 3-MA, in addition to its ability to inhibit class III PI3-kinase and associated autophagy, it may also inhibit class I PI3-kinases that regulate various cellular signaling such as membrane trafficking and mTORC1 activation (Mizushima et al., 2010). Taking this into consideration, high doses of 3-MA may produce adverse effects that are not dependent on autophagy, thereby impairing tubular cell viability. In this regard, Kim et al. used 1.5 times higher dosage of 3-MA than we did. Furthermore, they showed only an early but transient induction of autophagy (within 1 week) in the rat proximal tubules, whereas autophagy remained persistently activated for 6 months after RLDC treatment in our study. Pertaining to this, Li et al. showed that sustained autophagy in proximal tubules may inhibit tubular proliferation and repair during the recovery phase (Li et al., 2014). The pro-fibrotic role of tubular autophagy was also demonstrated by the study of Baisantry et al. (Baisantry et al., 2016), in which proximal tubule autophagy-related 5 (Atg5) knockout mice showed less interstitial fibrosis and improved renal function 30 days after ischemia/reperfusion injury. In contrast, Li et al. reported that selective ablation of Atg5 in proximal renal tubules significantly aggravated G2/M arrest, COL1 production, and fibrosis in UUO mice (Li et al., 2016). Although both studies tested Atg5 knockout mice, they examined renal fibrosis in different disease models. Therefore, the role of autophagy in renal fibrosis and chronic kidney disease may depend on the experimental model, animal species, and the timing or duration of autophagy activation.

For the mechanism whereby autophagy promotes renal fibrosis, we hypothesized that autophagy may promote a pro-fibrotic phenotype in renal tubular cells, which produce and secrete pro-fibrotic cytokines. To initially test this possibility, in this study we examined representative pro-fibrotic cytokines, such as TGFB, CTGF, and PDGFB. The expression of these pro-fibrotic growth factors was significantly increased after RLDC treatment (Figure 6). Notably, autophagy inhibition attenuated the production of TGFB and CTGF, but not PDGFB. This observation indicates that, rather than general effects, autophagy promotes specific pro-fibrotic cytokines. In addition, we observed that the two autophagy inhibitors, CQ and 3-MA, had different effects on TGFB and CTGF. For example, the inhibitory effect of 3-MA on CTGF is stronger than that of CQ, and it further affects the secretion of CTGF. The stronger effect of 3-MA may be due to its synergistic actions. It is known that, some PI3 kinase inhibitors, in addition to inhibiting autophagosome formation, may also inhibit mTOR by targeting its ATP-binding site (Mizushima et al., 2010). Furthermore, in addition to renal tubular cells, autophagy-regulated production of pro-fibrotic secretory proteins has also been demonstrated in fibroblasts. For instance, in a model of chronic serum starvation of fibroblasts, sustained autophagy activates MTORC2 signaling, resulting in enhanced expression and secretion of CTGF, which favors the transformation of fibroblasts into myofibroblasts (Bernard et al., 2014).

It remains unclear how autophagy promotes the expression and secretion of specific pro-fibrotic factors. As eluded above, we speculate that persistent autophagy may lead to phenotypic changes in renal tubular cells. Especially, autophagy may promote cell cycle arrest, metabolic shift, and/or senescence, which are known to contribute to maladaptive kidney repair including interstitial fibrosis (Venkatachalam et al., 2015; Zheng et al., 2019; Tang et al., 2020; Kitada and Koya, 2021). In a recent study, Canaud and others demonstrated the formation of mTOR-autophagy spatial coupling compartments (TASCCs), which promoted the secretion of profibrotic factors during maladaptive kidney repair (Canaud et al., 2019). Future studies should examine the effect of autophagy blockade on cell cycle arrest, senescence, and the formation of TASCCs after RLDC treatment. One limitation of this study is the possible off-target effects of the autophagy inhibitors. As mentioned above, 3-MA may also inhibit the class I PI3-kinases and, in turn, regulate various cellular signaling such as membrane trafficking and mTORC1 activation (Mizushima et al., 2010). In addition to interfering with autophagic flux, CQ in endosomes/lysosomes also inhibits post-translational modification of newly synthesized proteins in endoplasmic reticulum or trans-Golgi network vesicles (e.g, glyco-syltransferases and proteases involved in the post translational processing that requires low pH) (Yoon et al., 2010; Ferreira et al., 2021). In addition, CQ could activate the transcriptional response of p53 to induce the activation of p53-regulated genes such as apoptotic gene targets (pig3 and bax) (Ferreira et al., 2021). In view of these off-target effects, we tested both 3-MA and CQ in the present study. Since these two inhibitors had similar inhibitory effects on renal fibrosis in post-RLDC kidneys, the results provide relatively reliable evidence for the pro-fibrotic role of autophagy in this model. We also tested rapamycin (1 mg/kg) as autophagy inducer in a pilot experiment, which did not have significant effects on renal function or Masson’s staining of renal fibrosis after RLDC treatment in mice. But, rapamycin reduced the expression of some fibrosis proteins like fibronectin and vimentin. Our explanation is that this effect of rapamycin was due to its inhibition of mTOR and related protein synthesis, and not due to its effect on autophagy.

In conclusion, in this study we have detected persistent autophagy activation in both *in vivo* and *in vitro* RLDC models of maladaptive kidney repair after cisplatin nephrotoxicity. We have further demonstrated that inhibition of autophagy improved kidney repair and attenuated renal interstitial fibrosis in these models, suggesting a critical role of autophagy in the development of chronic kidney problems after cisplatin nephrotoxicity. The pro-fibrotic function of autophagy is associated with its coordinated regulation of tubular cell death and the production and secretion of pro-fibrotic cytokines. Targeting autophagy may provide a new strategy to reduce renal fibrosis to prevent the progression of related renal diseases including CKD.

## Chemical Compounds

Chemical compounds studied in this article: Cisplatin (PubChem CID: 5702198); Chloroquine (PubChem CID: 2719); 3-Methyladenine (PubChem CID: 135398661).

## Data Availability

The original contributions presented in the study are included in the article/Supplementary Material, further inquiries can be directed to the corresponding author.

## Abbreviations

AKI, acute kidney injury; ATG, autophagy-related gene; *α*-SMA, alpha-smooth muscle actin; BSA, bovine serum albumin; BUMPT, Boston University mouse proximal tubular cell line; CKD, chronic kidney disease; CQ, chloroquine; COL-1, collagen type I; CTGF, connective tissue growth factor; DMEM, Dulbecco’s modified eagle medium; ECM, extracellular matrix; FN, fibronectin; GFR, glomerular filtration rate; HE, hematoxylin-eosin staining; IHC, immunohistochemistry; IF, immunofluorescence; mTOR, mammalian target of rapamycin; PDGF, platelet-derived growth factor; RLDC, repeated low dose cisplatin; SDS, sodium dodecyl sulfate; Scr, serum creatinine; TGF-β, transforming growth factor-β; TASCC, mTOR-autophagy spatial coupling compartments; VIM, vimentin; 3-MA, 3-methyladenine.

## References

[B1] Baisantry A. Bhayana S. Rong S. Ermeling E. Wrede C. Hegermann J. (2016). Autophagy Induces Prosenescent Changes in Proximal Tubular S3 Segments. J. Am. Soc. Nephrol. 27 (6), 1609–1616. 10.1681/ASN.2014111059 26487561 PMC4884098

[B2] Basile D. Bonventre J. Mehta R. Nangaku M. Unwin R. Rosner M. (2016). Progression after AKI: Understanding Maladaptive Repair Processes to Predict and Identify Therapeutic Treatments. J. Am. Soc. Nephrol. 27 (3), 687–697. 10.1681/ASN.2015030309 26519085 PMC4769207

[B3] Bennis Y. Savry A. Rocca M. Gauthier-Villano L. Pisano P. Pourroy B. (2014). Cisplatin Dose Adjustment in Patients with Renal Impairment, Which Recommendations Should We Follow? Int. J. Clin. Pharm. 36 (2), 420–429. 10.1007/s11096-013-9912-7 24435159

[B4] Bernard M. Dieudé M. Yang B. Hamelin K. Underwood K. Hébert M. (2014). Autophagy Fosters Myofibroblast Differentiation through MTORC2 Activation and Downstream Upregulation of CTGF. Autophagy 10 (12), 2193–2207. 10.4161/15548627.2014.981786 25495560 PMC4502773

[B5] Black L. Lever J. Traylor A. Chen B. Yang Z. Esman S. (2018). Divergent Effects of AKI to CKD Models on Inflammation and Fibrosis. Am. J. Physiol. Ren. Physiol. 315 (4), F1107–F18. 10.1152/ajprenal.00179.2018 PMC6230746 29897282

[B6] Canaud G. Brooks C. R. Kishi S. Taguchi K. Nishimura K. Magassa S. (2019). Cyclin G1 and TASCC Regulate Kidney Epithelial Cell G-M Arrest and Fibrotic Maladaptive Repair. Sci. Transl. Med. 11 (476), eaav4754. 10.1126/scitranslmed.aav4754 30674655 PMC6527117

[B7] Choi M. (2020). Autophagy in Kidney Disease. Annu. Rev. Physiol. 82, 297–322. 10.1146/annurev-physiol-021119-034658 31640469

[B8] Deng Z. Sun M. Wu J. Fang H. Cai S. An S. (2021). SIRT1 Attenuates Sepsis-Induced Acute Kidney Injury via Beclin1 Deacetylation-Mediated Autophagy Activation. Cell death Dis. 12 (2), 217. 10.1038/s41419-021-03508-y 33637691 PMC7910451

[B9] Ding Y. Kim S. Lee S. Koo J. Wang Z. Choi M. (2014). Autophagy Regulates TGF-β Expression and Suppresses Kidney Fibrosis Induced by Unilateral Ureteral Obstruction. J. Am. Soc. Nephrol. 25 (12), 2835–2846. 10.1681/ASN.2013101068 24854279 PMC4243349

[B10] Ellery S. Cai X. Walker D. Dickinson H. Kett M. (2015). Transcutaneous Measurement of Glomerular Filtration Rate in Small Rodents: through the Skin for the Win? Nephrol. Carlt. Vic. 20 (3), 117–123. 10.1111/nep.12363 25388805

[B11] Ferenbach D. Bonventre J. (2015). Mechanisms of Maladaptive Repair after AKI Leading to Accelerated Kidney Ageing and CKD. Nat. Rev. Nephrol. 11 (5), 264–276. 10.1038/nrneph.2015.3 25643664 PMC4412815

[B12] Ferreira P. Sousa R. Ferreira J. Militão G. Bezerra D. (2021). Chloroquine and Hydroxychloroquine in Antitumor Therapies Based on Autophagy-Related Mechanisms. Pharmacol. Res. 168, 105582. 10.1016/j.phrs.2021.105582 33775862

[B13] Forbes M. Thornhill B. Chevalier R. (2011). Proximal Tubular Injury and Rapid Formation of Atubular Glomeruli in Mice with Unilateral Ureteral Obstruction: a New Look at an Old Model. Am. J. Physiol. Ren. Physiol. 301 (1), F110–F117. 10.1152/ajprenal.00022.2011 PMC3129891 21429968

[B14] Fu Y. Cai J. Li F. Liu Z. Shu S. Wang Y. (2019). Chronic Effects of Repeated Low-Dose Cisplatin Treatment in Mouse Kidneys and Renal Tubular Cells. Am. J. Physiol. Ren. Physiol. 317 (6), F1582–F92. 10.1152/ajprenal.00385.2019 31532246

[B15] Geng H. Lan R. Wang G. Siddiqi A. Naski M. Brooks A. (2009). Inhibition of Autoregulated TGFbeta Signaling Simultaneously Enhances Proliferation and Differentiation of Kidney Epithelium and Promotes Repair Following Renal Ischemia. Am. J. Pathol. 174 (4), 1291–1308. 10.2353/ajpath.2009.080295 19342372 PMC2671361

[B16] Grgic I. Campanholle G. Bijol V. Wang C. Sabbisetti V. Ichimura T. (2012). Targeted Proximal Tubule Injury Triggers Interstitial Fibrosis and Glomerulosclerosis. Kidney Int. 82 (2), 172–183. 10.1038/ki.2012.20 22437410 PMC3480325

[B17] Humphreys B. Cantaluppi V. Portilla D. Singbartl K. Yang L. Rosner M. (2016). Targeting Endogenous Repair Pathways after AKI. J. Am. Soc. Nephrol. 27 (4), 990–998. 10.1681/ASN.2015030286 26582401 PMC4814191

[B18] Jiang M. Wei Q. Dong G. Komatsu M. Su Y. Dong Z. (2012). Autophagy in Proximal Tubules Protects against Acute Kidney Injury. Kidney Int. 82 (12), 1271–1283. 10.1038/ki.2012.261 22854643 PMC3491167

[B19] Kim S. Na H. Ding Y. Wang Z. Lee S. Choi M. (2012). Autophagy Promotes Intracellular Degradation of Type I Collagen Induced by Transforming Growth Factor (TGF)-β1. J. Biol. Chem. 287 (15), 11677–11688. 10.1074/jbc.M111.308460 22351764 PMC3320917

[B20] Kim W. Nam S. Song H. Ko J. Park S. Kim H. (2012). The Role of Autophagy in Unilateral Ureteral Obstruction Rat Model. Nephrol. Carlt. Vic. 17 (2), 148–159. 10.1111/j.1440-1797.2011.01541.x 22085202

[B21] Kitada M. Koya D. (2021). Autophagy in Metabolic Disease and Ageing. Nat. Rev. Endocrinol. 17 (11), 647–661. 10.1038/s41574-021-00551-9 34508250

[B22] Klionsky D. Abdel-Aziz A. Abdelfatah S. Abdellatif M. Abdoli A. Abel S. (2021). Guidelines for the Use and Interpretation of Assays for Monitoring Autophagy (4th Edition). Autophagy 17 (1), 1–382. 10.1080/15548627.2015.1100356 33634751 PMC7996087

[B23] Koesters R. Kaissling B. Lehir M. Picard N. Theilig F. Gebhardt R. (2010). Tubular Overexpression of Transforming Growth Factor-Beta1 Induces Autophagy and Fibrosis but Not Mesenchymal Transition of Renal Epithelial Cells. Am. J. Pathol. 177 (2), 632–643. 10.2353/ajpath.2010.091012 20616344 PMC2913362

[B24] Kok H. Falke L. Goldschmeding R. Nguyen T. (2014). Targeting CTGF, EGF and PDGF Pathways to Prevent Progression of Kidney Disease. Nat. Rev. Nephrol. 10 (12), 700–711. 10.1038/nrneph.2014.184 25311535

[B25] Lan R. Geng H. Polichnowski A. Singha P. Saikumar P. McEwen D. (2012). PTEN Loss Defines a TGF-β-Induced Tubule Phenotype of Failed Differentiation and JNK Signaling during Renal Fibrosis. Am. J. Physiol. Ren. Physiol. 302 (9), F1210–F1223. 10.1152/ajprenal.00660.2011 PMC3362177 22301622

[B26] Landau S. Guo X. Velazquez H. Torres R. Olson E. Garcia-Milian R. (2019). Regulated Necrosis and Failed Repair in Cisplatin-Induced Chronic Kidney Disease. Kidney Int. 95 (4), 797–814. 10.1016/j.kint.2018.11.042 30904067 PMC6543531

[B27] Lee S. Kim S. Choi M. (2015). Therapeutic Targets for Treating Fibrotic Kidney Diseases. Transl. Res. J. lab. Clin. Med. 165 (4), 512–530. 10.1016/j.trsl.2014.07.010 PMC4326607 25176603

[B28] Li H. Peng X. Wang Y. Cao S. Xiong L. Fan J. (2016). Atg5-mediated Autophagy Deficiency in Proximal Tubules Promotes Cell Cycle G2/M Arrest and Renal Fibrosis. Autophagy 12 (9), 1472–1486. 10.1080/15548627.2016.1190071 27304991 PMC5082781

[B29] Li L. Wang Z. Hill J. Lin F. (2014). New Autophagy Reporter Mice Reveal Dynamics of Proximal Tubular Autophagy. J. Am. Soc. Nephrol. JASN. 25 (2), 305–315. 10.1681/ASN.2013040374 24179166 PMC3904563

[B30] Li L. Zepeda-Orozco D. Black R. Lin F. (2010). Autophagy Is a Component of Epithelial Cell Fate in Obstructive Uropathy. Am. J. pathology 176 (4), 1767–1778. 10.2353/ajpath.2010.090345 PMC2843468 20150430

[B31] Liu B. Tang T. Lv L. Lan H. (2018). Renal Tubule Injury: a Driving Force toward Chronic Kidney Disease. Kidney Int. 93 (3), 568–579. 10.1016/j.kint.2017.09.033 29361307

[B32] Liu J. Livingston M. Dong G. Tang C. Su Y. Wu G. (2018). Histone Deacetylase Inhibitors Protect against Cisplatin-Induced Acute Kidney Injury by Activating Autophagy in Proximal Tubular Cells. Cell death Dis. 9 (3), 322. 10.1038/s41419-018-0374-7 29476062 PMC5833747

[B33] Livingston M. Ding H. Huang S. Hill J. Yin X. Dong Z. (2016). Persistent Activation of Autophagy in Kidney Tubular Cells Promotes Renal Interstitial Fibrosis during Unilateral Ureteral Obstruction. Autophagy 12 (6), 976–998. 10.1080/15548627.2016.1166317 27123926 PMC4922446

[B34] Livingston M. Wang J. Zhou J. Wu G. Ganley I. Hill J. (2019). Clearance of Damaged Mitochondria via Mitophagy Is Important to the Protective Effect of Ischemic Preconditioning in Kidneys. Autophagy 15 (12), 2142–2162. 10.1080/15548627.2019.1615822 31066324 PMC6844514

[B35] Miller R. Tadagavadi R. Ramesh G. Reeves W. (2010). Mechanisms of Cisplatin Nephrotoxicity. Toxins 2 (11), 2490–2518. 10.3390/toxins2112490 22069563 PMC3153174

[B36] Mizushima N. Yoshimori T. Levine B. (2010). Methods in Mammalian Autophagy Research. Cell 140 (3), 313–326. 10.1016/j.cell.2010.01.028 20144757 PMC2852113

[B37] Periyasamy-Thandavan S. Jiang M. Wei Q. Smith R. Yin X. Dong Z. (2008). Autophagy Is Cytoprotective during Cisplatin Injury of Renal Proximal Tubular Cells. Kidney Int. 74 (5), 631–640. 10.1038/ki.2008.214 18509315

[B38] Sears S. Siskind L. (2021). Potential Therapeutic Targets for Cisplatin-Induced Kidney Injury: Lessons from Other Models of AKI and Fibrosis. J. Am. Soc. Nephrol. 32 (7), 1559–1567. 10.1681/asn.2020101455 34049962 PMC8425641

[B39] Sharp C. Doll M. Megyesi J. Oropilla G. Beverly L. Siskind L. (2018). Subclinical Kidney Injury Induced by Repeated Cisplatin Administration Results in Progressive Chronic Kidney Disease. Am. J. Physiol. Ren. Physiol. 315 (1), F161–F72. 10.1152/ajprenal.00636.2017 PMC6087791 29384415

[B40] Shi M. McMillan K. Wu J. Gillings N. Flores B. Moe O. (2018). Cisplatin Nephrotoxicity as a Model of Chronic Kidney Disease. Lab. Invest. a J. Tech. Methods Pathol. 98 (8), 1105–1121. 10.1038/s41374-018-0063-2 PMC6528473 29858580

[B41] Shu S. Wang H. Zhu J. Liu Z. Yang D. Wu W. (2021). Reciprocal Regulation between ER Stress and Autophagy in Renal Tubular Fibrosis and Apoptosis. Cell death Dis. 12 (11), 1016. 10.1038/s41419-021-04274-7 34716302 PMC8556380

[B42] Skinner R. Parry A. Price L. Cole M. Craft A. Pearson A. (2009). Persistent Nephrotoxicity during 10-year Follow-Up after Cisplatin or Carboplatin Treatment in Childhood: Relevance of Age and Dose as Risk Factors. Eur. J. cancer 45 (18), 3213–3219. 10.1016/j.ejca.2009.06.032 19850470

[B43] Tang C. Han H. Yan M. Zhu S. Liu J. Liu Z. (2018). PINK1-PRKN/PARK2 Pathway of Mitophagy Is Activated to Protect against Renal Ischemia-Reperfusion Injury. Autophagy 14 (5), 880–897. 10.1080/15548627.2017.1405880 29172924 PMC6070003

[B44] Tang C. Livingston M. Liu Z. Dong Z. (2020). Autophagy in Kidney Homeostasis and Disease. Nat. Rev. Nephrol. 16 (9), 489–508. 10.1038/s41581-020-0309-2 32704047 PMC7868042

[B45] Torres R. Velazquez H. Chang J. Levene M. Moeckel G. Desir G. (2016). Three-Dimensional Morphology by Multiphoton Microscopy with Clearing in a Model of Cisplatin-Induced CKD. J. Am. Soc. Nephrol. JASN 27 (4), 1102–1112. 10.1681/ASN.2015010079 26303068 PMC4814184

[B46] Venkatachalam M. Weinberg J. Kriz W. Bidani A. (2015). Failed Tubule Recovery, AKI-CKD Transition, and Kidney Disease Progression. J. Am. Soc. Nephrol. 26 (8), 1765–1776. 10.1681/ASN.2015010006 25810494 PMC4520181

[B47] Wang Y. Tang C. Cai J. Chen G. Zhang D. Zhang Z. (2018). PINK1/Parkin-mediated Mitophagy Is Activated in Cisplatin Nephrotoxicity to Protect against Kidney Injury. Cell death Dis. 9 (11), 1113. 10.1038/s41419-018-1152-2 30385753 PMC6212494

[B48] Xu Y. Ruan S. Wu X. Chen H. Zheng K. Fu B. (2013). Autophagy and Apoptosis in Tubular Cells Following Unilateral Ureteral Obstruction Are Associated with Mitochondrial Oxidative Stress. Int. J. Mol. Med. 31 (3), 628–636. 10.3892/ijmm.2013.1232 23314838

[B49] Yoon Y. Cho K. Hwang J. Lee S. Choi J. Koh J. (2010). Induction of Lysosomal Dilatation, Arrested Autophagy, and Cell Death by Chloroquine in Cultured ARPE-19 Cells. Invest. Ophthalmol. Vis. Sci. 51 (11), 6030–6037. 10.1167/iovs.10-5278 20574031

[B50] Zheng K. He Z. Kitazato K. Wang Y. (2019). Selective Autophagy Regulates Cell Cycle in Cancer Therapy. Theranostics 9 (1), 104–125. 10.7150/thno.30308 30662557 PMC6332805

