# Corrigendum: Persistent activation of autophagy after cisplatin nephrotoxicity promotes renal fibrosis and chronic kidney disease

**DOI:** 10.3389/fphar.2024.1387592

**Published:** 2024-03-06

**Authors:** Ying Fu, Yu Xiang, Wenwen Wu, Juan Cai, Chengyuan Tang, Zheng Dong

**Affiliations:** ^1^ Department of Nephrology, Hunan Key Laboratory of Kidney Disease and Blood Purification, The Second Xiangya Hospital at Central South University, Changsha, China; ^2^ Department of Cellular Biology and Anatomy, Medical College of Georgia at Augusta University and Charlie Norwood VA Medical Center, Augusta, GA, United States

**Keywords:** autophagy, cisplatin, kidney injury and repair, renal fibrosis, profibrotic growth factor

In the published article, there was an error in Figure 2E as published. The unit of measurement is incorrectly listed as “uL/min/100 g b.w.” and should be corrected to “mL/min/100 g b.w.”. The corrected Figure 2 and its caption Pharmacologic inhibition of autophagy alleviates renal dysfunction and tubular damage in post-RLDC kidneys appear below.

**FIGURE 2:**
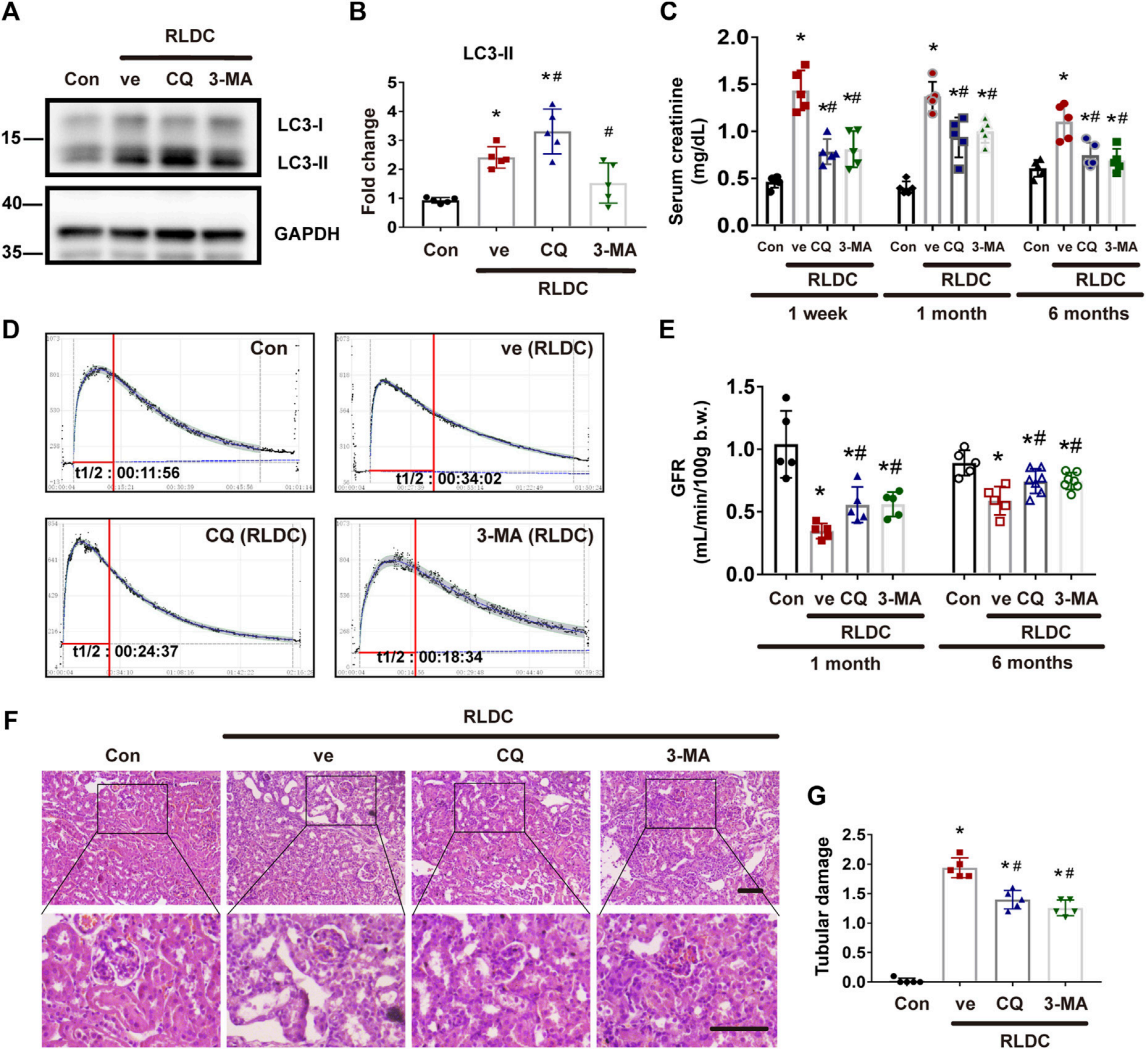
Pharmacologic inhibition of autophagy alleviates renal dysfunction and tubular damage in post-RLDC kidneys. Male C57BL/6 mice were injected weekly with 8 mg/kg cisplatin for 4 weeks (RLDC) or with saline as control (Con). After the final dose, the mice were injected with 60 mg/kg/day chloroquine (CQ), 20 mg/kg/day 3-methyladenine (3-MA), or saline as vehicle solution (ve) for 7 days. **(A)** Representative immunoblots of LC3-I, LC3-II and GAPDH (loading control) in kidney tissues (*n* = 5). **(B)** Densitometry of LC3II. The experiments were normalized according to GAPDH expression. The protein level of control group (Con) was arbitrarily set as 1, and the signals of other conditions were normalized with the control group to indicate their protein fold changes. **(C)** Effect of autophagy inhibitor on serum creatinine at 1 week, 1 month and 6 months after RLDC treatment. (*n* = 5). **(D)** Representative tracing curves of FITC-sinistrin clearance in mice. (*n* = 5). **(E)** GFR measurement by transcutaneously monitoring FITC-sinistrin clearance (*n* = 5). **(F)** Representative histology images of hematoxylin-eosin staining of kidney tissues in renal cortex and outer medulla. (*n* = 5, bar = 50 μm). **(G)** Pathological score of tubular damage. Quantitative data are expressed as mean ± SEM. *p < 0.05 vs the control group (Con), # *p* < 0.05 vs. (RLDC + vehicle) group.

The authors apologize for this error and state that this does not change the scientific conclusions of the article in any way. The original article has been updated.

