# Supplementary figures and images for: Persistent Activation of Autophagy After Cisplatin Nephrotoxicity Promotes Renal Fibrosis and Chronic Kidney Disease
Source: Front Pharmacol. 2022 May 30;13:918732. doi: 10.3389/fphar.2022.918732 (PMC9189407; doi:10.3389/fphar.2022.918732)

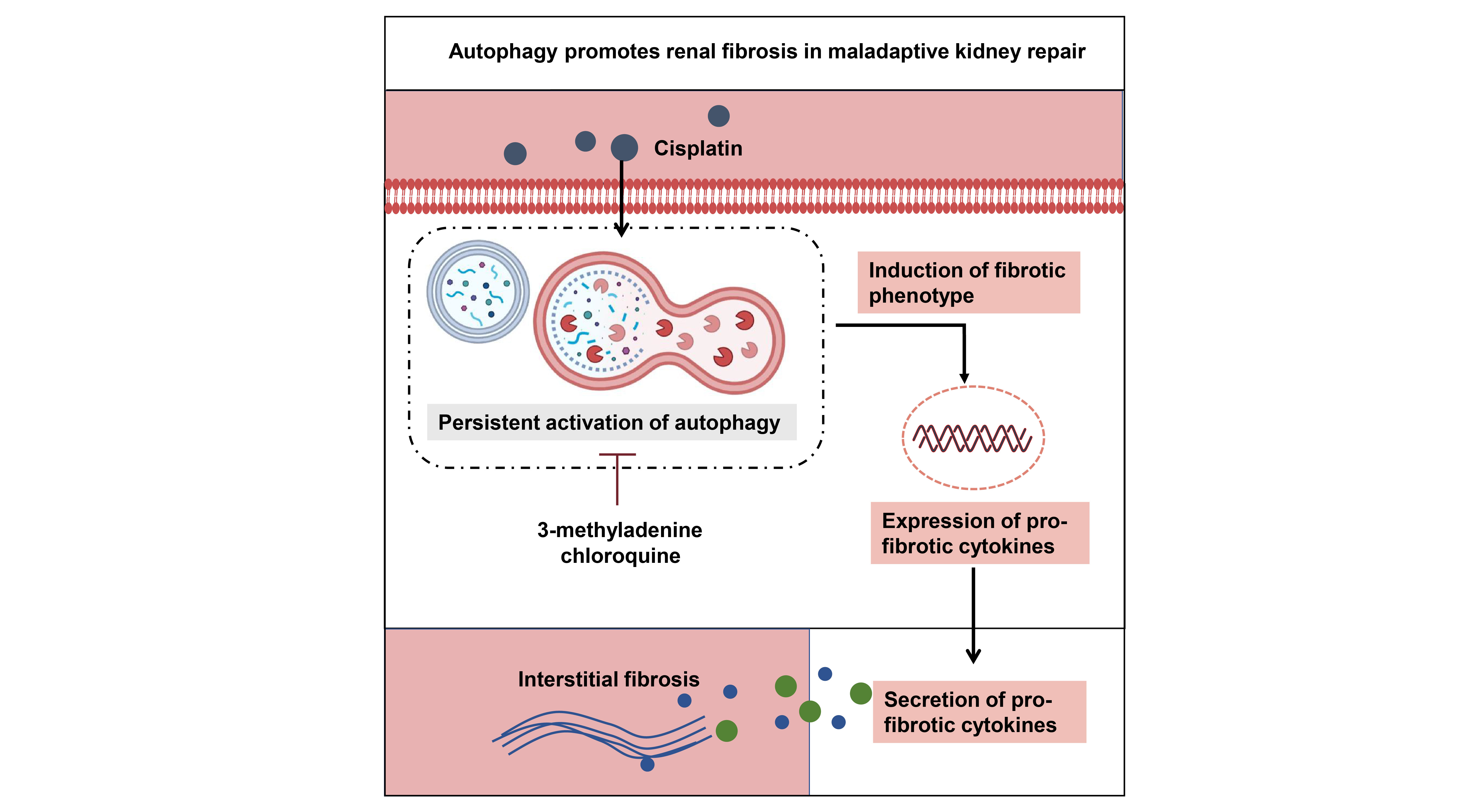

Supplement: Supplementary file 1 [file Image1.JPEG]
