# Supplementary material for: Persistent Activation of Autophagy After Cisplatin Nephrotoxicity Promotes Renal Fibrosis and Chronic Kidney Disease
Source: Front Pharmacol. 2022 May 30;13:918732. doi: 10.3389/fphar.2022.918732 (PMC9189407; doi:10.3389/fphar.2022.918732)

Figure 1D LC3B (NOVUS; NB100-2220)
Collagen I (Affinity; AF7001)
GAPDH(Proteintech; 10494-1-AP)

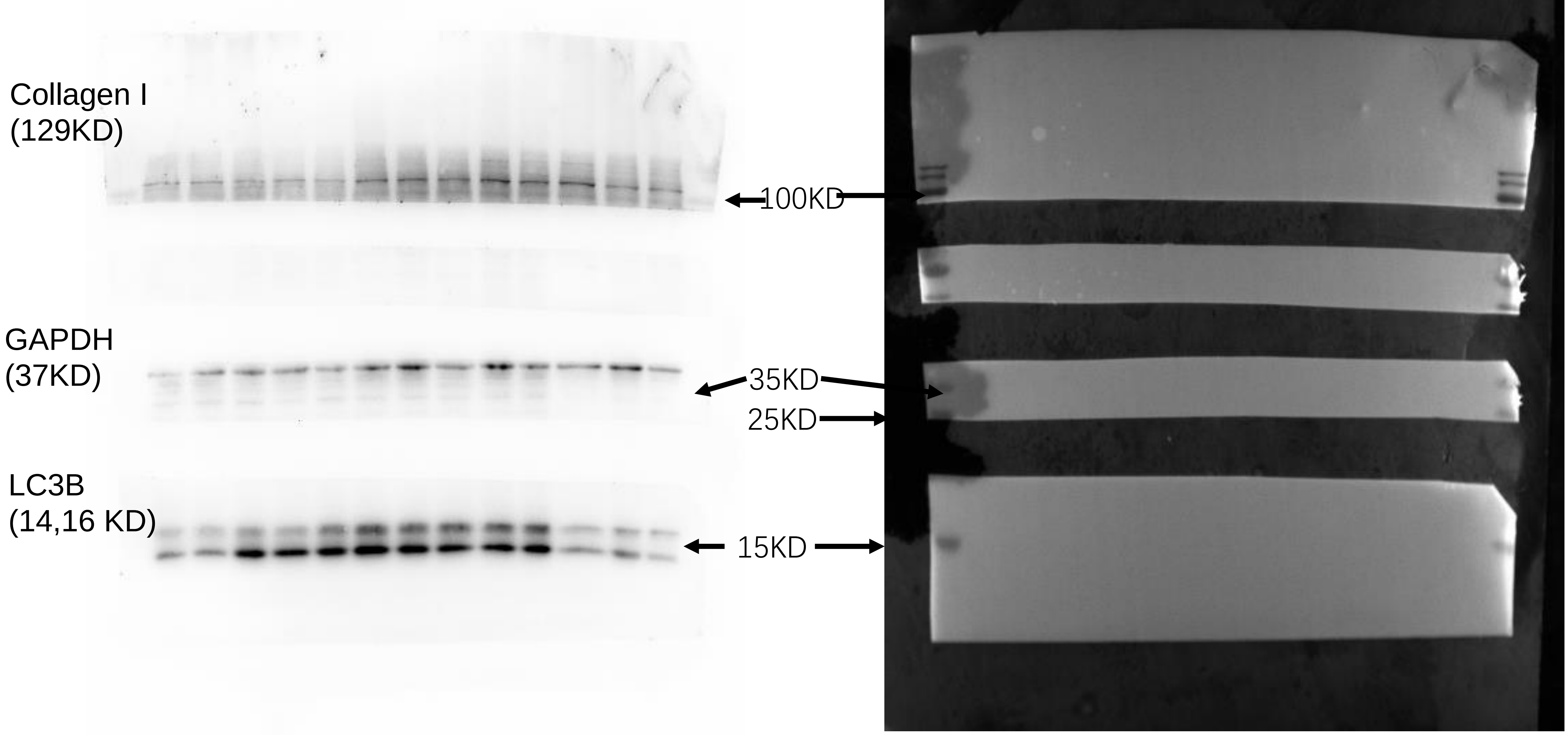

Figure 2A LC3B (NOVUS; NB100-2220)
GAPDH(Proteintech; 10494-1-AP)

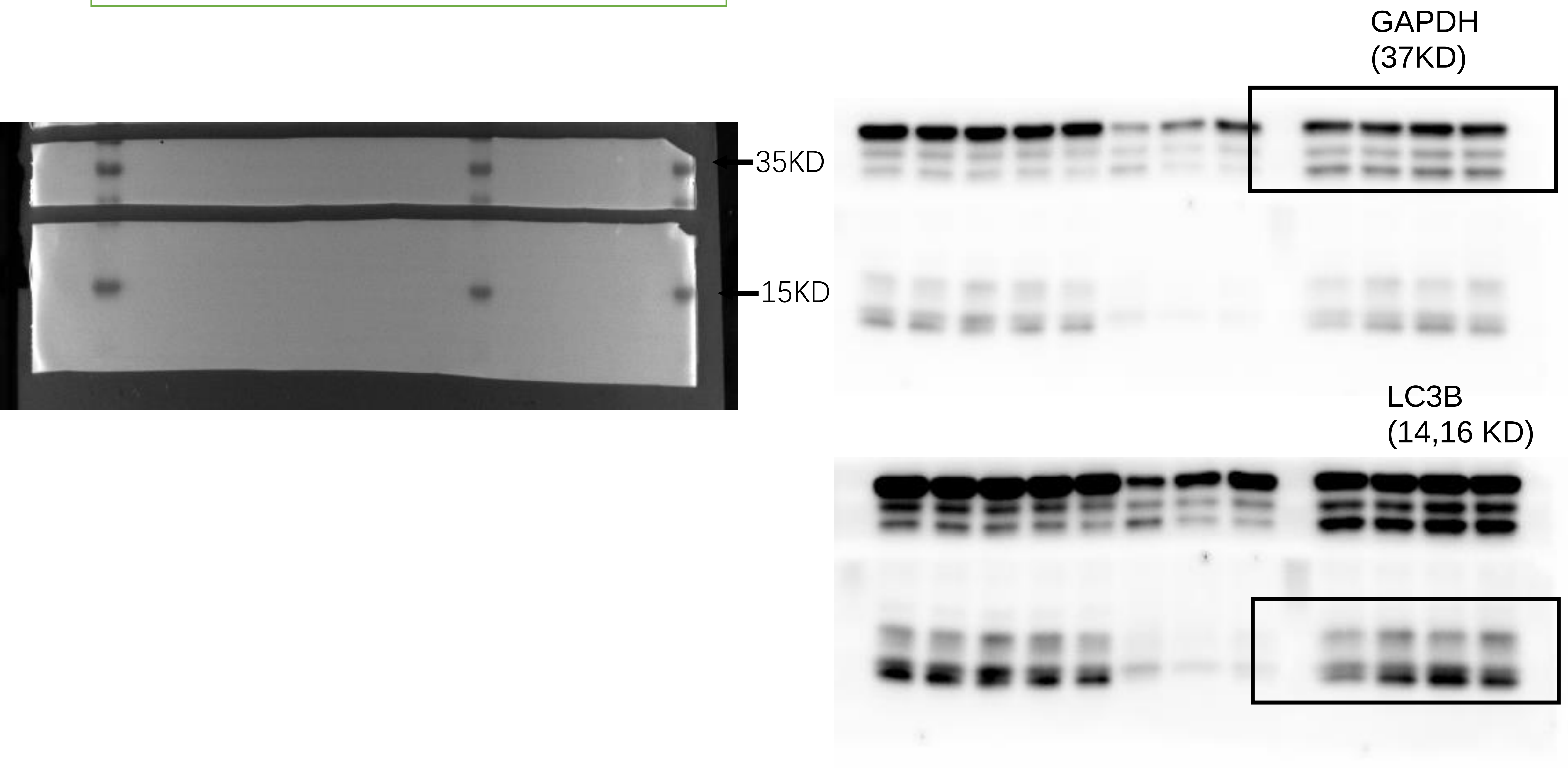

Figure 3A
FN (abcam; ab2413;
Vimentin (CST; 5741)
GAPDH(Proteintech; 10494-1-AP)
Anti-alpha smooth muscle Actin antibody (abcam; ab5694)

FN
(262KD)

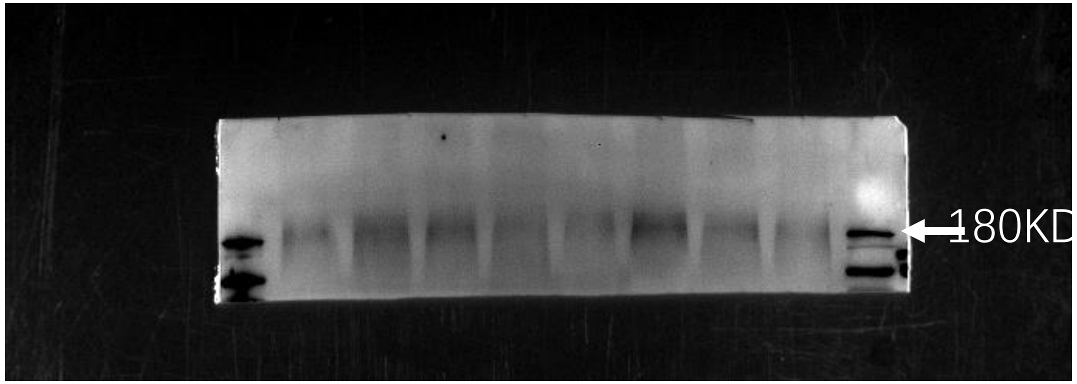

GA
(37KD)

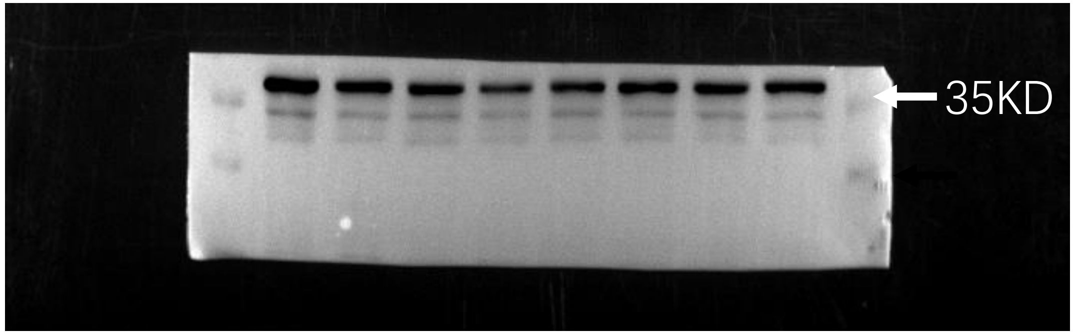

VIM
(55KD)

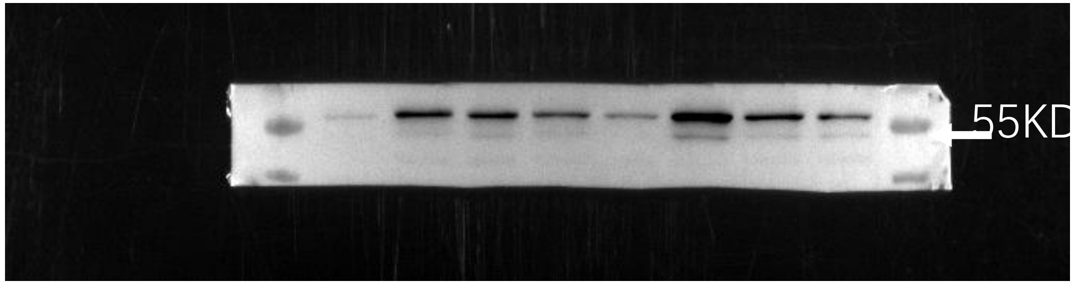

alpha-
SMA
(42KD)

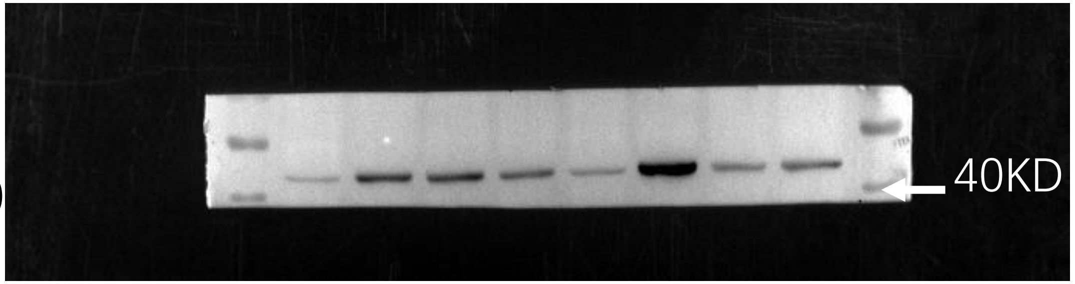

Figure 4A
FN (abcam; ab2413;)
LC3B (NOVUS; NB100-2220)
GAPDH(Proteintech; 10494-1-AP)
Collagen I (Affinity; AF7001)
Cleaved Caspase-3 (Asp175) (CST; 9661)

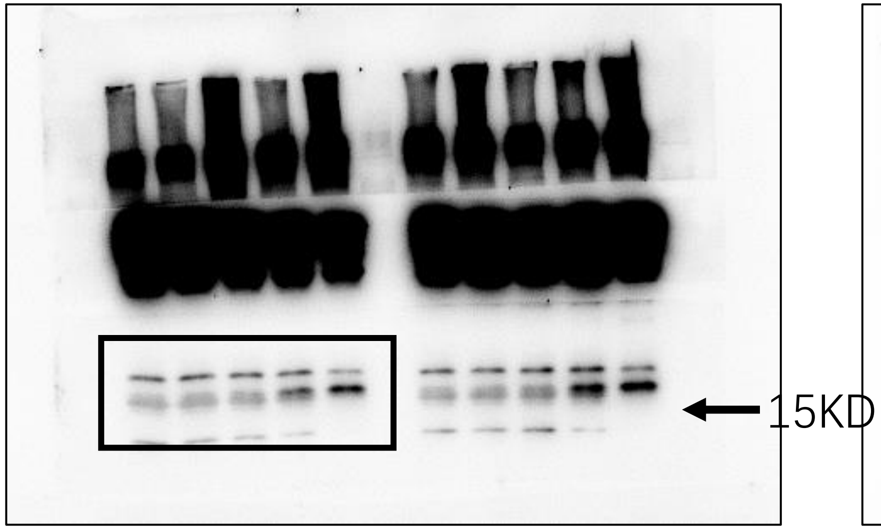

c-cas3 (17,19 KD)

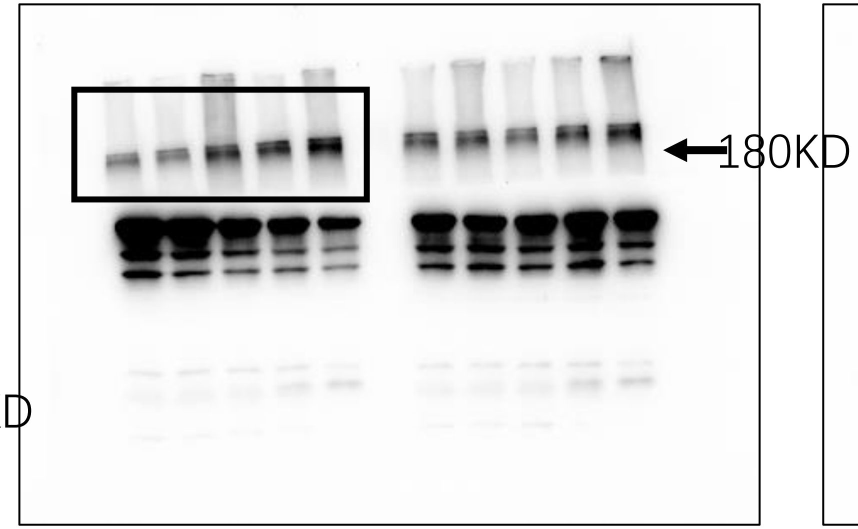

FN (262KD)

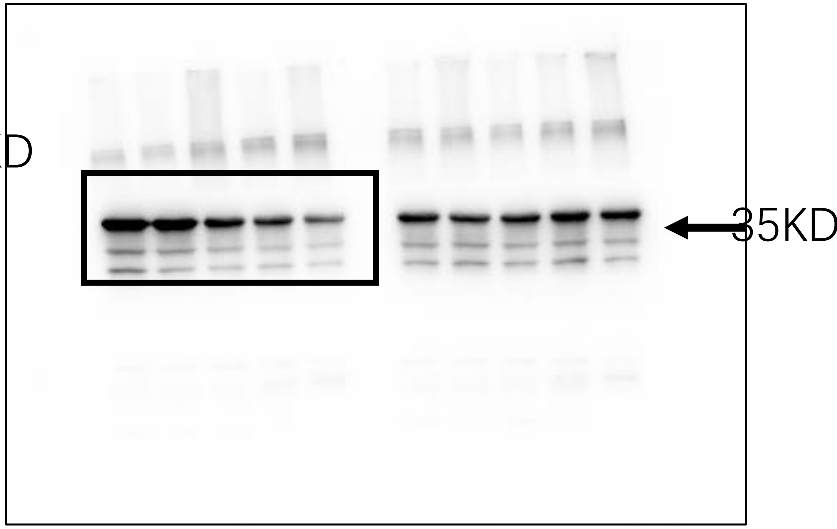

GA (37KD)

Collagen I
(129KD)

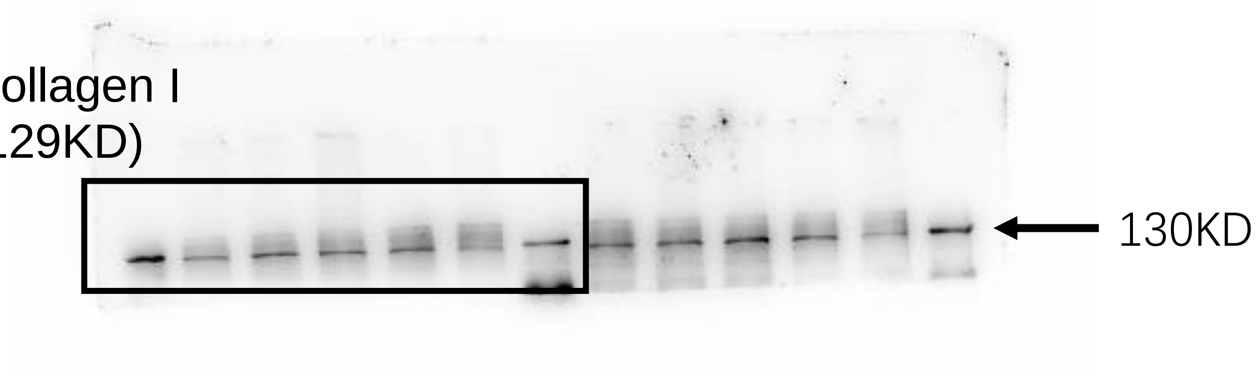

Figure 4C
FN (abcam; ab2413;)
LC3B (NOVUS; NB100-2220)
GAPDH(Proteintech; 10494-1-AP)

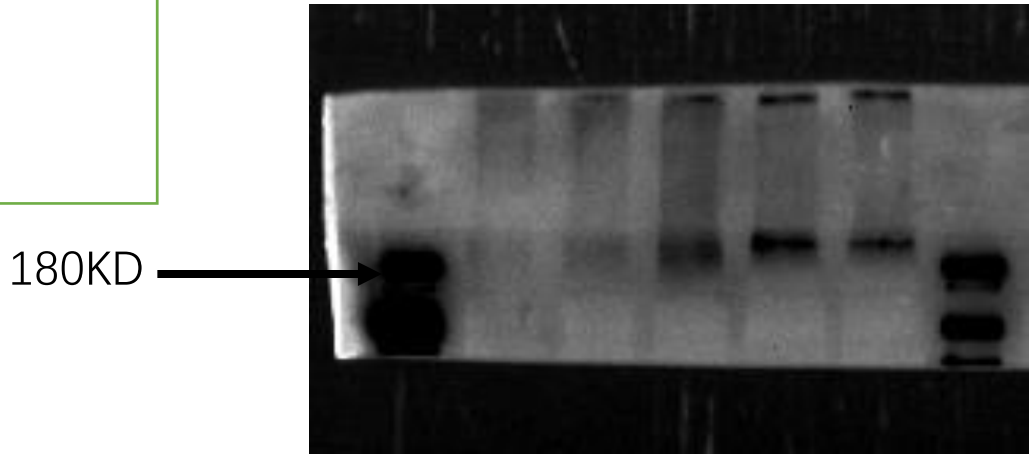

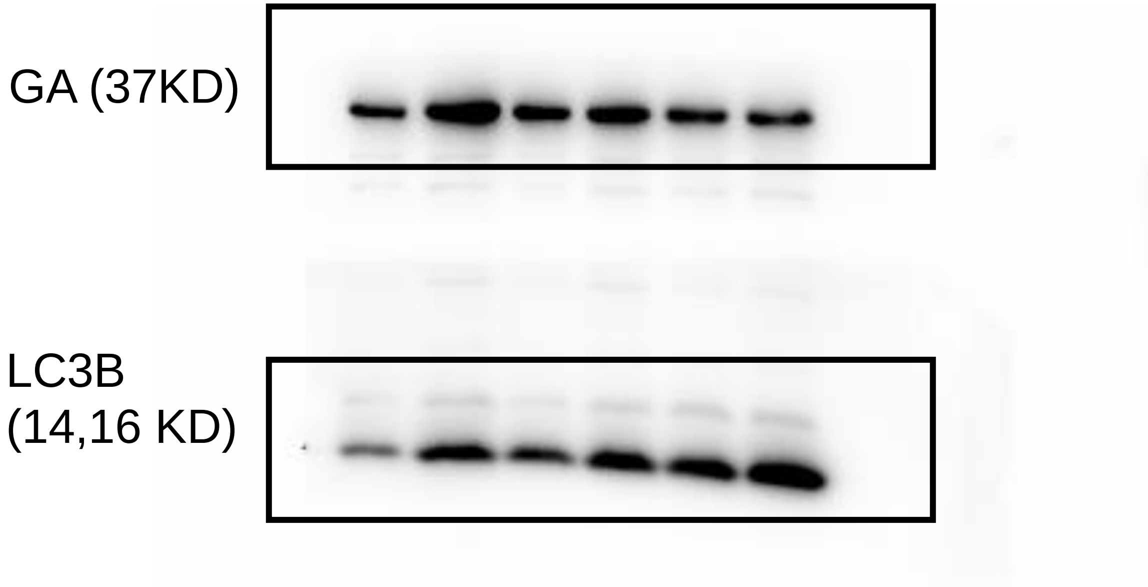

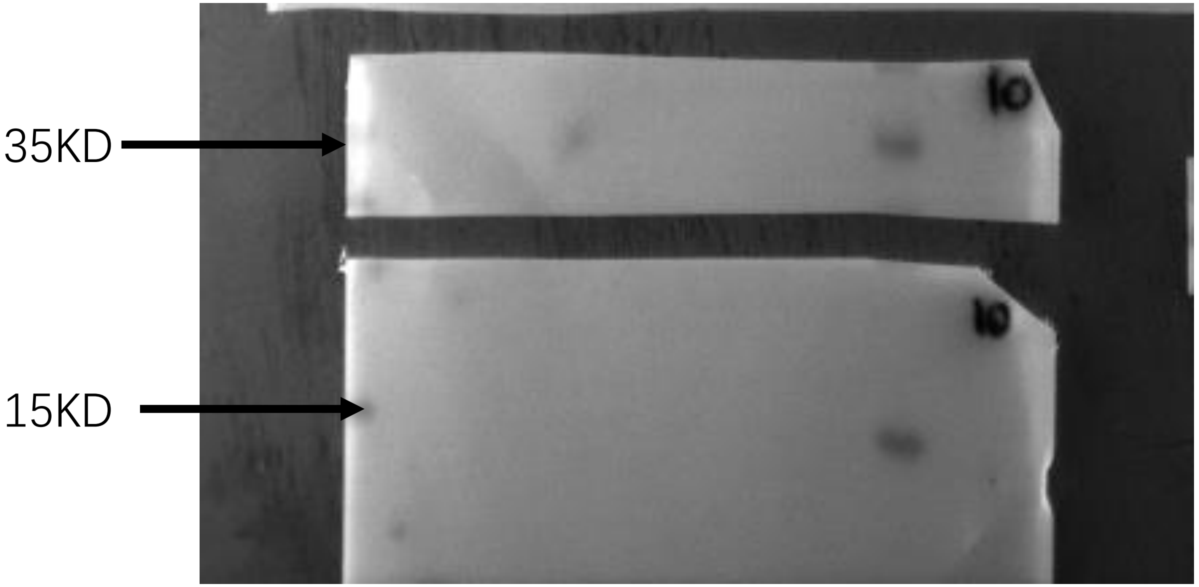

Figure 5B
Collagen I (Affinity; AF7001)
LC3B (NOVUS; NB100-2220)
Vimentin (CST; 5741)
GAPDH(Proteintech; 10494-1-AP)

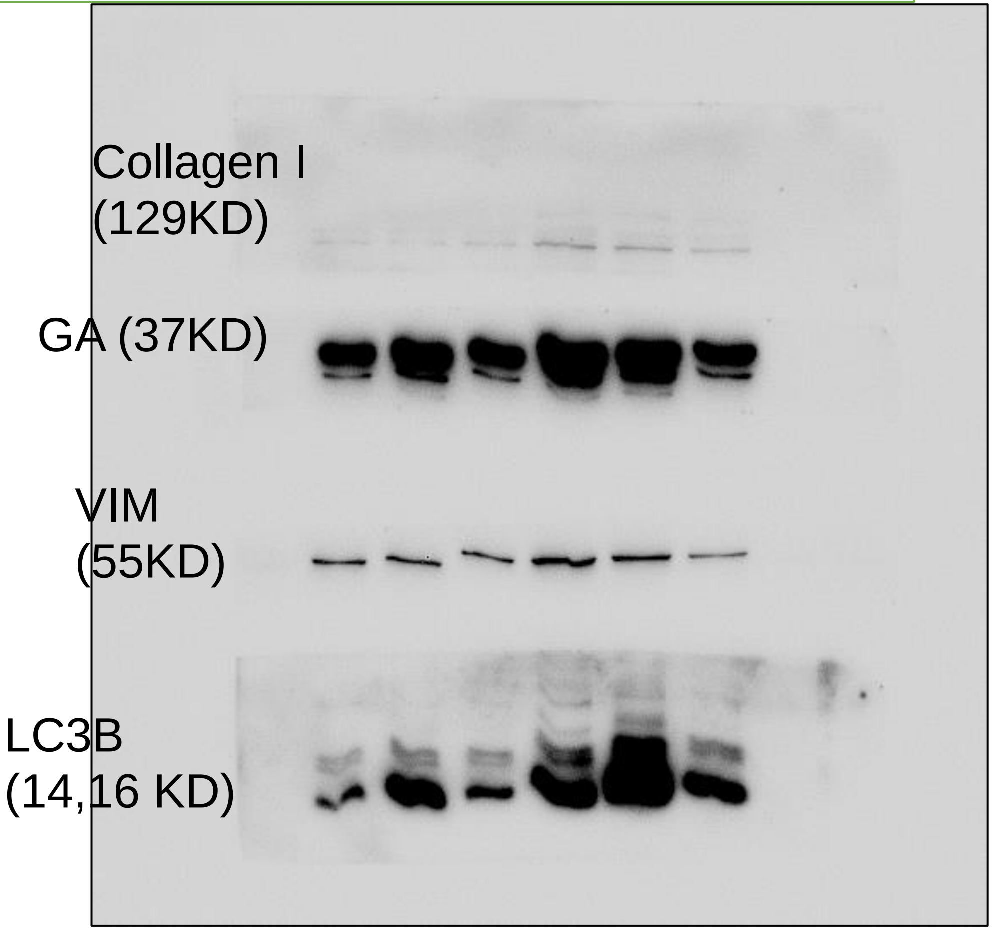

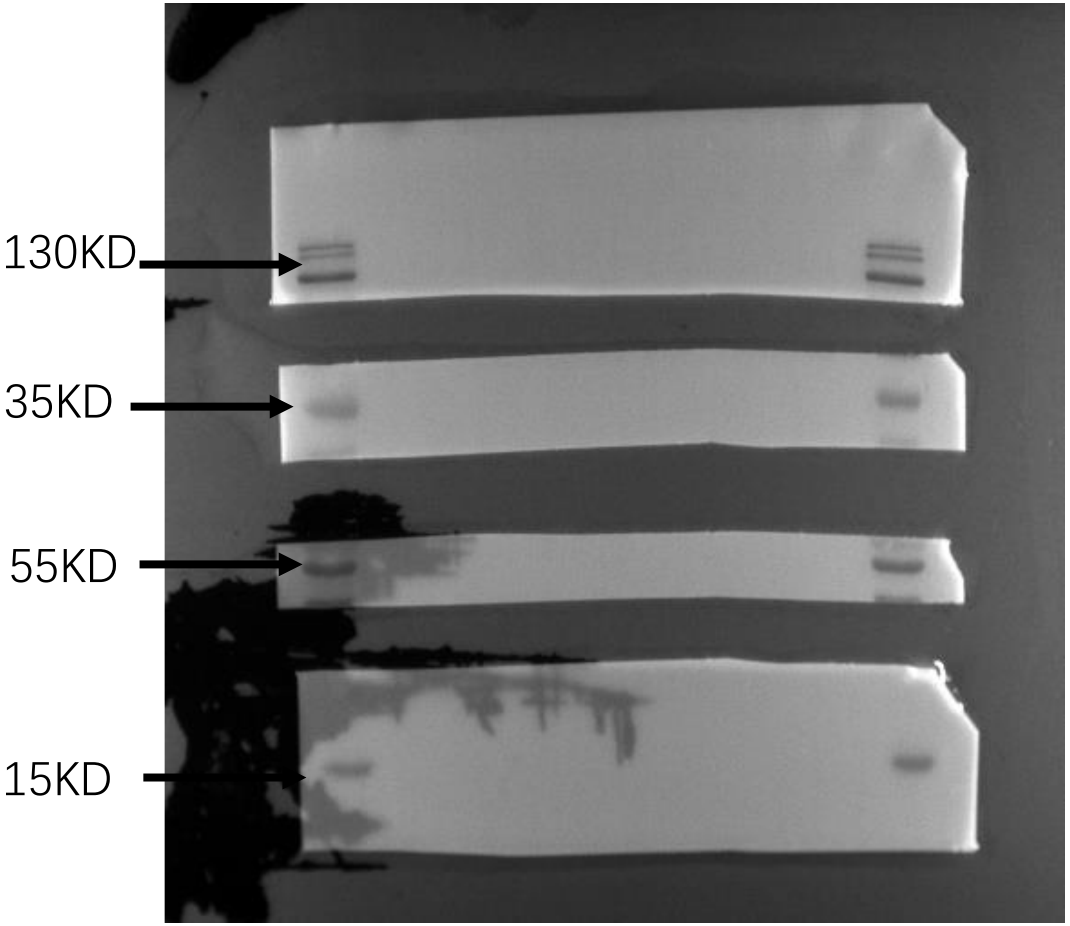

Supplement: Supplementary file 2 [file DataSheet1.PDF]
